# In Situ Observations of Interstellar Pickup Ions from 1 au to the Outer Heliosphere

**DOI:** 10.1007/s11214-022-00895-2

**Published:** 2022-05-09

**Authors:** E. J. Zirnstein, E. Möbius, M. Zhang, J. Bower, H. A. Elliott, D. J. McComas, N. V. Pogorelov, P. Swaczyna

**Affiliations:** 1 Department of Astrophysical Sciences, Princeton University, Princeton, NJ 08544 USA; 2 Space Science Center and Department of Physics, University of New Hampshire, Durham, NH 03824 USA; 3 Department of Physics and Space Sciences, Florida Institute of Technology, Melbourne, FL 32901 USA; 4 Space Science and Engineering Division, Southwest Research Institute, San Antonio, TX 78228 USA; 5 Department of Physics and Astronomy, University of Texas at San Antonio, San Antonio, TX 78249 USA; 6 Department of Space Science, The University of Alabama in Huntsville, Huntsville, AL 35805 USA; 7 Center for Space Plasma and Aeronomic Research, The University of Alabama in Huntsville, Huntsville, AL 35805 USA

**Keywords:** Pickup ion, Heating, Acceleration, Heliosphere, Interstellar medium, Interstellar neutrals

## Abstract

Interstellar pickup ions are an ubiquitous and thermodynamically important component of the solar wind plasma in the heliosphere. These PUIs are born from the ionization of the interstellar neutral gas, consisting of hydrogen, helium, and trace amounts of heavier elements, in the solar wind as the heliosphere moves through the local interstellar medium. As cold interstellar neutral atoms become ionized, they form an energetic ring beam distribution comoving with the solar wind. Subsequent scattering in pitch angle by intrinsic and self-generated turbulence and their advection with the radially expanding solar wind leads to the formation of a filled-shell PUI distribution, whose density and pressure relative to the thermal solar wind ions grows with distance from the Sun.

This paper reviews the history of in situ measurements of interstellar PUIs in the heliosphere. Starting with the first detection in the 1980s, interstellar PUIs were identified by their highly nonthermal distribution with a cutoff at twice the solar wind speed. Measurements of the PUI distribution shell cutoff and the He focusing cone, a downwind region of increased density formed by the solar gravity, have helped characterize the properties of the interstellar gas from near-Earth vantage points. The preferential heating of interstellar PUIs compared to the core solar wind has become evident in the existence of suprathermal PUI tails, the nonadiabatic cooling index of the PUI distribution, and PUIs’ mediation of interplanetary shocks. Unlike the Voyager and Pioneer spacecraft, New Horizon’s Solar Wind Around Pluto (SWAP) instrument is taking the only direct measurements of interstellar PUIs in the outer heliosphere, currently out to $\sim47~\text{au}$ from the Sun or halfway to the heliospheric termination shock.

## Introduction

As our solar system moves through the interstellar medium, the solar wind plasma emanating from the Sun and moving at supersonic speeds interacts with the partially-ionized, local interstellar medium, forming the heliosphere (Parker 1961). The interstellar medium surrounding our heliosphere is composed primarily of H and He neutral and ionized components co-moving at $\sim25~\text{km}\,\text{s}^{-1}$ with respect to our solar system. While the interstellar ionized plasma is slowed and diverted around the outer boundary of the heliosphere (Baranov and Malama 1993), i.e., the heliopause, interstellar neutral particles propagate into the heliosphere and can become ionized in the solar wind through a variety of ionization processes (Blum and Fahr 1970; Bzowski et al. 2013; Ruciński et al. 1996; Sokół et al. 2020; Wallis 1971, 1975; Zank 2015, 1999). Once an interstellar neutral atom becomes ionized, it is “picked up” by the magnetic and motional electric field of the solar wind plasma, which is moving much faster than the interstellar neutrals in the solar inertial frame. This results in the production and incorporation of interstellar pickup ions (PUIs) at highly non-thermal speeds in the reference frame of the solar wind (Isenberg 1986; Vasyliunas and Siscoe 1976).

The number density of PUIs in the solar wind depends on the local neutral density, the rate of neutral ionization, and the accumulation of PUIs closer to the Sun that are advected outwards with the radially propagating solar wind. $\text{He}^{+}$ PUIs dominate the PUI source near Earth due to their high ionization potential and ability for interstellar He atoms to propagate closer to the Sun before experiencing ionization (Axford 1972; Siscoe and Mukherjee 1972). Beyond a few au, however, $\text{H}^{+}$ PUIs become the dominant PUI species. Far from the Sun ($>20~\text{au}$), interstellar $\text{H}^{+}$ PUIs constitute the majority of the internal pressure of the solar wind plasma (McComas et al. 2017b), and already account for more than 10% of the proton number density halfway to the heliospheric termination shock (HTS) (McComas et al. 2021).

After their discovery (Möbius et al. 1985b), a suite of spacecraft in the heliosphere have provided a wealth of interstellar PUI measurements spanning nearly four decades. These measurements have revealed both the ubiquitous presence of PUIs in the solar wind but also their diagnostic capabilities at inferring properties of the local interstellar medium (Gloeckler et al. 2004; Gloeckler and Geiss 2001a; Möbius et al. 1995). Due to their large range of phase velocities compared to colder solar wind ions (SWIs) released from the solar corona, measurements have shown that PUIs experience preferential heating at interplanetary shocks (Gloeckler et al. 2000b, 1994a; Gloeckler and Geiss 1998; Starkey et al. 2021; Zirnstein et al. 2018). Interplanetary shocks in the outer heliosphere become, on average, increasingly quasi-perpendicular farther from the Sun owing to the Parker-spiral nature of the interplanetary magnetic field (IMF) (Parker 1958), where the IMF orientation is nearly perpendicular to the bulk solar wind velocity and normal of the shock surface. Numerous theoretical and modeling studies have proposed mechanisms responsible for the preferential acceleration of PUIs at shocks and the development of suprathermal tails at energies higher than the PUI injection speed (Burrows et al. 2010; Giacalone et al. 2021, 1994; Kumar et al. 2018; Lee et al. 1996; Lipatov et al. 1998; Perri et al. 2022; Yang et al. 2015; Zank et al. 1996; Zilbersher and Gedalin 1997; Zirnstein et al. 2021b). As the interstellar PUI number density relative to the SWIs grows with distance from the Sun (Lee et al. 2009), their internal pressure downstream of shocks dominates the internal pressure of the bulk solar wind plasma itself and thus clearly play a significant role in mediating shocks in the outer heliosphere (McComas et al. 2021).

Interstellar PUIs are preferentially accelerated at the HTS, which surrounds our solar system, resulting in a high-beta plasma in the inner heliosheath dominated by accelerated PUIs. While there has yet to be any direct observation of PUI acceleration at the HTS, it can be inferred from Voyager 2 observations of the core SWIs. Observations by the PLS instrument revealed that the SWIs were not subsonic downstream of the shock, where $\sim 80\%$ of the energy dissipated by the slowing of the solar wind at the shock appeared to be missing (Richardson et al. 2008a). It is generally agreed that the PUIs, which are not directly observable by Voyager spacecraft, hold the bulk of the remaining energy (Zank et al. 2010).

The best evidence of energetic PUIs in the outer heliosphere beyond the HTS is from remote energetic neutral atom (ENA) observations by the Interstellar Boundary Explorer (IBEX) (McComas et al. 2009b), which reveal power-law like ENA spectra across the sky at energies between $\sim0.5$ and 6 keV (Dayeh et al. 2014, 2011; Desai et al. 2019, 2012; Galli et al. 2022; McComas et al. 2020, 2017a, 2009a; Zirnstein et al. 2021a, 2020). ENA emissions from the inner heliosheath originate from interstellar PUIs in the solar wind that were preferentially accelerated at the HTS and advected downstream (Kumar et al. 2018; Sokół et al. 2022; Yang et al. 2015; Zank et al. 2010; Zirnstein et al. 2021b). SOHO HSTOF and Cassini INCA also observe ENAs from the heliosheath, although at higher energies (Czechowski et al. 2020; Dialynas et al. 2022, 2017; Hilchenbach et al. 1998; Krimigis et al. 2009; Westlake et al. 2020).

This review focuses on direct, in situ observations of interstellar PUIs by spacecraft in the heliosphere. In the following sections, we review in situ observations of interstellar PUIs measured between 1 and $\sim47~\text{au}$ from the Sun, including measurements from AMPTE-IRM SULEICA, ACE SWICS, SOHO CELIAS, and STEREO PLASTIC near 1 au, Ulysses SWICS between $\sim1$ and 5 au, Cassini CAPS and CHEMS out to $\sim10~\text{au}$, and New Horizons’ SWAP from $\sim10$ to $\sim47~\text{au}$. These instruments utilize a variety of measurement techniques to observe the PUI distribution in the heliosphere, revealing insightful information about PUI speed distributions, the contributions of PUIs to the solar wind plasma pressure, and preferential PUI acceleration at interplanetary shocks, including the development of suprathermal tails. Radial trends of PUI density and pressure relative to the bulk solar wind plasma are compared across multiple data sets. Extrapolations of interstellar PUI properties to the HTS can be used to assess their contribution to the inner heliosheath plasma pressure and ENA fluxes observed by remote imagers.

## Observations of Interstellar PUIs in the Inner Heliosphere

The first direct observation of interstellar PUIs was that of $\text{He}^{+}$ near Earth. From the discovery of interstellar $\text{He}^{+}$ PUIs, they were recognized as an essential tool to obtain the physical parameters of the interstellar neutral gas from inside the heliosphere and as a critical source population for further acceleration (Möbius et al. 1985b). In this section, we review the initial discovery of interstellar PUIs and the subsequent measurements made by multiple spacecraft in the inner heliosphere (within $\sim10~\text{au}$ of the Sun).

### Detection and Early Findings

With the realization that interstellar neutral gas flows freely through the heliosphere (Fahr 1968) instead of being kept out almost entirely like in a stellar Strömgren Sphere (Strömgren 1939), it became apparent that interstellar gas reaches the inner heliosphere and interacts with the solar wind through different ionization processes (Axford 1972; Fahr 1974; Holzer 1972; Thomas 1972). Scattering of solar UV radiation off interstellar neutral atoms made them detectable, leading to the first information about the physical parameters of interstellar H (Adams and Frisch 1977; Bertaux and Blamont 1971; Thomas and Krassa 1971) and He (Paresce et al. 1973; Weller and Meier 1974). Solar UV, charge exchange with SWIs, and electron impact progressively ionize the interstellar gas as it approaches the Sun. This situation generated substantial interest in the contribution of singly charged minor ions to the solar wind and the related interactions. Also, the role of electrostatic instabilities in incorporating the interstellar ions into the solar wind and its potential heating were discussed (Hartle and Wu 1973).

Interstellar H atoms are swiftly ionized in the heliosphere compared to other neutral particles and most of them become PUIs before they can reach Earth’s orbit. Therefore, thanks to its high ionization potential, He is the primary source of newly injected PUIs at 1 au (Axford 1972; Siscoe and Mukherjee 1972; Sokół et al. 2020, 2019a). Therefore, efforts started to detect interstellar $\text{He}^{+}$ in the solar wind population (Feldman et al. 1972b,a). However, besides an occasionally elevated $\text{He}^{+}$ abundance in coronal mass ejections (Bame et al. 1968; Schwenn et al. 1980), only upper limits for interstellar $\text{He}^{+}$ were found with solar wind instruments (Feldman et al. 1974). Motivated by this negative result, Vasyliunas and Siscoe (1976) suggested that newly generated PUIs likely maintain a broad suprathermal velocity distribution in the solar wind rather than being rapidly thermalized. According to the analytical solution by Vasyliunas and Siscoe (1976), the distribution function of PUIs in the solar wind frame as a function of speed $v$, radial distance $r$, and angle from the interstellar flow direction $\theta $ can be expressed as

1 $$ f_{\mathrm{PUI}} ( r, w,\theta ) = \frac{3}{8\pi} \frac{\beta _{0} r_{0}^{2}}{r V_{\mathrm{sw}} V_{\mathrm{inj}}^{3}} w^{-3/2} n ( r,w, \theta ) \Theta ( 1-w ), $$

where $w= {v} / {V_{\mathrm{inj}}}$, $v$ is the PUI speed in the solar wind frame, $V_{\mathrm{inj}}$ is the PUI injection/cutoff speed, $\beta _{0}$ is the ionization rate at $r_{0} =1~\text{au}$, $\lambda $ is the neutral source ionization cavity size, and $n ( r,w, \theta )$ is the density of neutral source particles to be ionized at a reduced radial distance $rw^{3/2}$ and angle $\theta $ from the interstellar neutral inflow direction. Such a broad distribution as defined by Eq. (1) would be undetectable for typical solar wind instruments of the day.

**Fig. 1:**
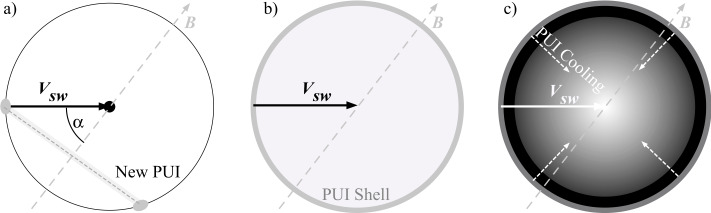
Cut through the PUI velocity distribution in the $\boldsymbol{V}_{\mathrm{sw}}$–$\boldsymbol{B}$ plane. (**a**) New PUIs form a ring around $\boldsymbol{B}$ on a spherical shell with radius $V_{\mathrm{sw}}$ at the pitch-angle $\alpha $. (**b**) Rapid pitch-angle scattering distributes the PUIs isotropically over this outermost velocity shell. (**c**) On a slower timescale, the radial expansion of the solar wind leads to adiabatic cooling of the PUIs, thus shrinking the shell, while newly injected PUIs continually fill the outermost shell at larger distances $r$ from the Sun. The final PUI velocity distribution fills a sphere around the core solar wind

Illustrating the PUI velocity distribution in Fig. 1, a newly ionized particle is injected into the solar wind with the velocity $-\boldsymbol{V}_{\mathrm{SW}}$, when the interstellar neutral gas flow is much slower than the solar wind. In the solar wind frame, the PUI starts to gyrate about the IMF at pitch angle $\alpha $ on a shell in velocity space with radius $V_{\mathrm{SW}}$ centered on the solar wind (Fig. 1a). It is typically assumed that IMF fluctuations cause rapid pitch-angle scattering, with the PUIs filling the outermost shell in velocity space (at $V_{\mathrm{SW}}$) isotropically (Fig. 1b) (Isenberg 1986; Isenberg and Lee 1996; Lee and Ip 1987; Möbius et al. 1988). Finally, the radial expansion of the solar wind acts on this isotropic shell velocity distribution like adiabatic cooling. In response, the shell shrinks toward the solar wind distribution at the center while being replaced by newly generated PUIs in the outermost shell. The result is a PUI phase-space distribution that fills the sphere in velocity space as $f(v < V_{\mathrm{SW}}) = f_{0}\cdot v^{\alpha -3}$ (a simplified form of the generalized distribution; Chen et al. (2013)), where $\alpha $ is the adiabatic cooling index. Assuming three degrees of freedom for the PUI distribution and a solar wind expansion as $1/r^{2}$ with the distance from the Sun, this index has been widely taken as $\alpha = 3/2$ after Vasyliunas and Siscoe (1976).

**Fig. 2:**
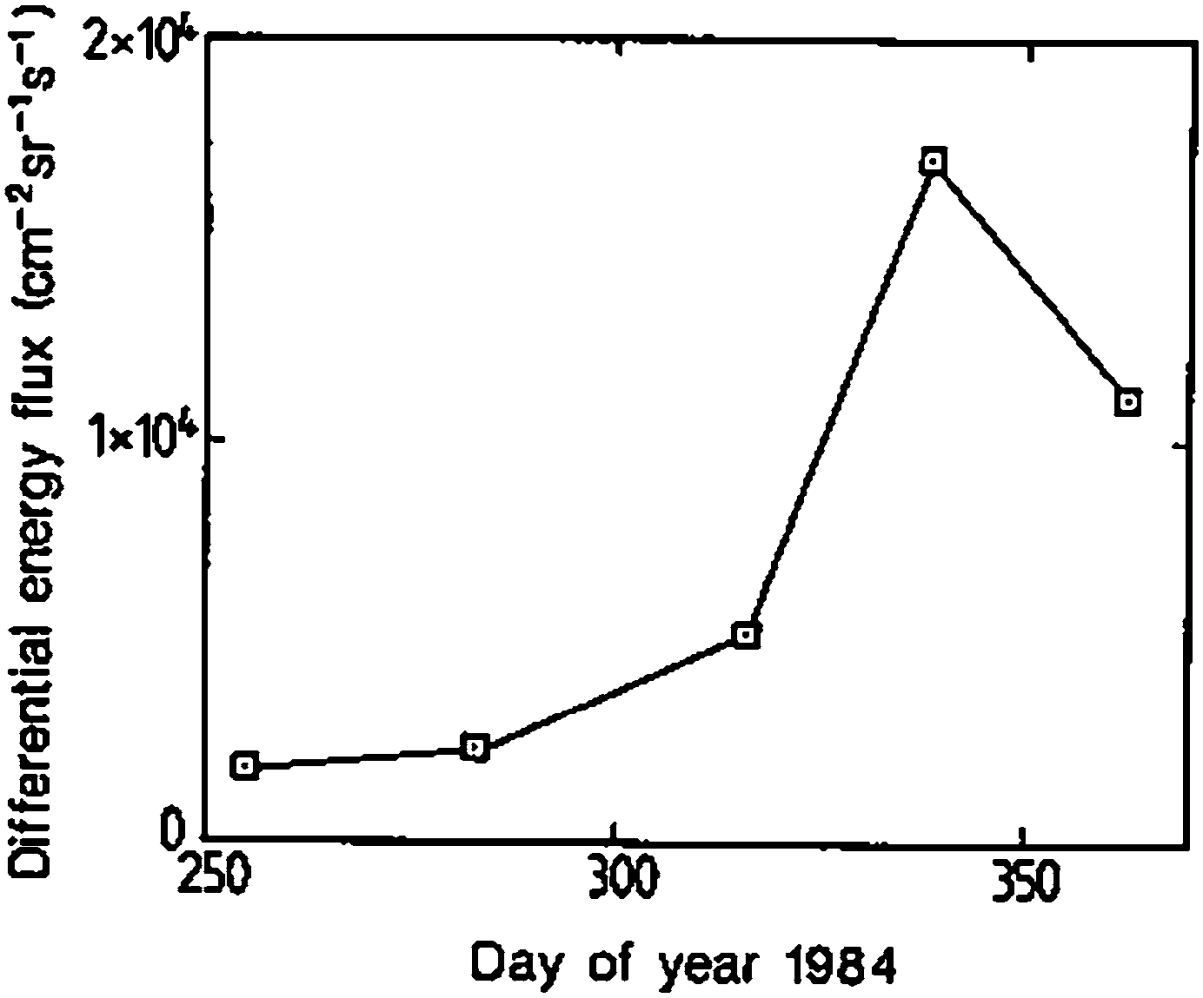
Differential energy flux of $\text{He}^{+}$ PUIs as measured by the SULEICA instrument onboard AMPTE-IRM at 20 keV as a function of time between September and December 1984. From Möbius et al. (1985b). Reproduced with permission from Springer Nature

The advent of sensitive space-borne time-of-flight (TOF) mass spectrographs in the 1980s for the suprathermal energy range (Gloeckler et al. 1985; Möbius et al. 1985a) provided a tool with the intrinsic capability to discriminate interstellar PUIs among the dominant solar wind. $\text{He}^{+}$ PUIs were observed among $\text{Li}^{+}$ PUIs injected into the solar wind (Möbius et al. 1986) during the first active ion cloud experiments with the AMPTE-IRM spacecraft (Haerendel et al. 1985). Möbius et al. (1985b) recognized the $\text{He}^{+}$ PUIs were of interstellar origin because of their broad velocity distribution, as predicted by Vasyliunas and Siscoe (1976). Also, their fluxes are more than three orders of magnitude above those expected from the exosphere (Fahr and Shizgal 1983), and the distinct temporal variation of the PUI fluxes with a maximum in early December mimics the gravitational focusing of the interstellar He gas flow (Fahr 1974), as shown in Fig. 2.

**Fig. 3:**
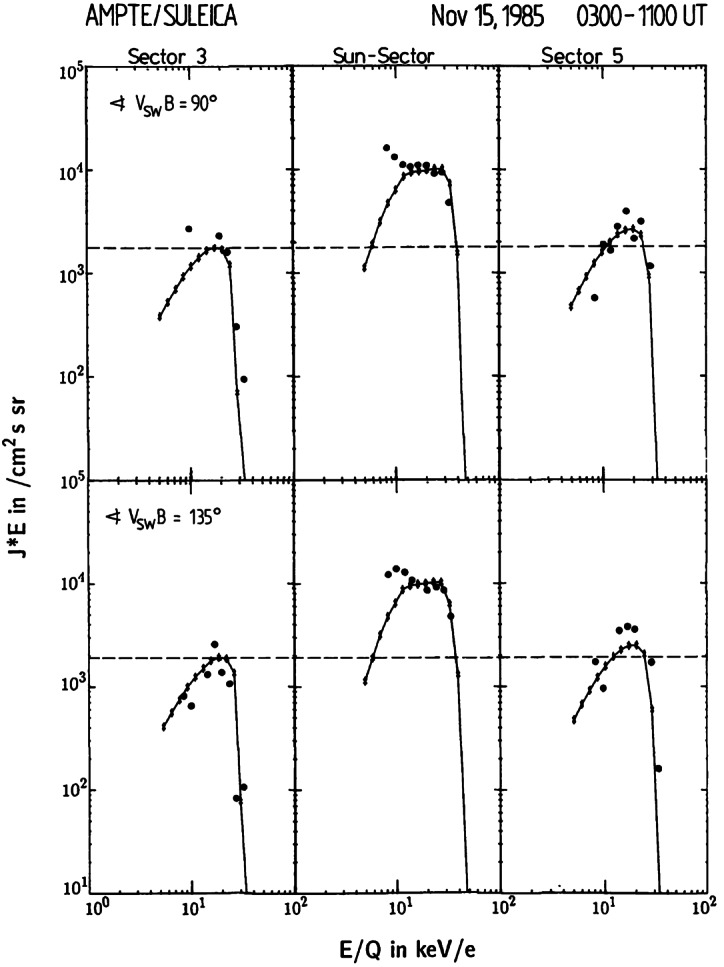
AMPTE-IRM SULEICA observations of interstellar $\text{He}^{+}$ PUIs (filled circles) from sectors pointed near the Sun. Measurements correspond to times when the solar wind speed $V_{\mathrm{SW}} = \sim680~\text{km}\,\text{s}^{-1}$ and the angle between the solar wind flow velocity and IMF was $\sim90^{\circ}$ and $\sim135^{\circ}$. Models for the PUI distribution functions following Vasyliunas and Siscoe (1976) are also shown (stars with solid curve). From Möbius et al. (1988). Reproduced with permission from Springer Nature

Figure 3 shows the first comparison between modeled PUI velocity distributions using the approach by Vasyliunas and Siscoe (1976) and the $\text{He}^{+}$ PUIs observed with the AMPTE-IRM SUprathermaL Energy Ion Charge Analyzer (SULEICA) instrument (Möbius et al. 1988). As can be seen, the predictions by the Vasyliunas and Siscoe (1976) model show relatively good agreement with the observations, including the presence of the sharp cut-off, the plateau at energies below the cut-off, and the difference in intensities in the different angular sectors. Because the PUI distribution showed a relatively sharp cut-off around $2V_{\mathrm{sw}}$ in the observer frame, diffusion in velocity space, also discussed as potentially important in the evolution of the PUI distribution (Isenberg 1987), appeared to have only a minor influence. We note, however, that there are few measurements below $\sim1~\text{keV}$ for this PUI data set, or in other words below the solar wind energy. One of the main reasons for this limitation is that for a given angular and energy resolution, the observed count rates from a PUI distribution decrease sharply toward lower energies.

Immediately with the discovery of the interstellar PUIs, it became evident that this ion population must be the feeder into the acceleration of the anomalous cosmic ray (ACR) component (Möbius et al. 1985b). The ACRs were thought to be of interstellar origin (Fisk 1976). It turned out that PUIs formed a velocity distribution that lends itself to substantially more effective injection into acceleration to higher energies than the bulk solar wind (Gloeckler et al. 1994a; Kucharek et al. 2003; Morris et al. 2001). Therefore, PUIs are a natural bridge between neutral source distributions, such as interstellar gas, and energetic particles such as ACRs (Giacalone et al. 2022).

### Instrumentation for PUI Measurements in the Inner Heliosphere

The advent of TOF spectrographs that allow species determination in the suprathermal energy range and provide superior background and noise suppression due to their built-in coincidence measurement in space physics enabled the diagnostics of PUIs in various environments (Möbius et al. 2016a; Wüest 1998). As mentioned in Sect. 2.1, the SULEICA instrument (Möbius et al. 1985a) on the AMPTE-IRM spacecraft (Häusler et al. 1985) ushered in the era of interstellar PUI diagnostics. SULEICA combines a spherical section electrostatic analyzer (ESA), a cylindrical section TOF spectrograph, and solid-state detectors (SSD) to determine the energy ($E$), atomic mass number ($A$), and ionic charge ($Q$) of the incoming ions. The ESA defines a $40^{\circ}\times 6^{\circ}$ instantaneous field-of-view (FOV), and the spacecraft spin covers $40^{\circ}\times 360^{\circ}$, centered on the ecliptic plane and divided into eight angular sectors. Incoming ions exit the ESA, selected for $E/Q$ with an energy per charge width $\Delta(E/Q)/(E/Q) = 0.1$ (FWHM), and then enter the TOF spectrograph, which determines the speed $v$ of the ion and thus $E/A$ through a TOF measurement. Combining both measurements, provides $A/Q = (E/Q)/(E/A)$. An SSD stops the ion and measures the energy $E$ of the ion. The combination of all three measurements yields a unique determination of $E$, $A$, and $Q$. Because most PUIs are singly charged and form a separate, very distinct velocity distribution, the ESA and TOF combination often suffices for the PUI analysis. The angular sectoring and logarithmically spaced steps in $E/Q$, covering 10–270 keV/Q, provide a two-dimensional cut through the PUI distribution in the ecliptic plane.

The Solar Wind Ion Composition Spectrograph (SWICS) on Ulysses and ACE (Gloeckler et al. 1998, 1992) is based on a similar combination of measurement techniques. To provide detailed measurements of the solar wind composition, SWICS features post-acceleration of the incoming ions after passing the ESA by 23 kV, boosting their energy by 23 keV/Q. The SWICS spherical sector ESA has an $80^{\circ}\times 10^{\circ}$ FOV. The near edge of the SWICS FOV is $10^{\circ}$ from the spin axis, thus providing a conical FOV with a half-width of $70^{\circ}$ that always includes the Sun. The SWICS data are sub-divided into eight spin-angle sectors. The ACE spin axis points within $6^{\circ}$ of the Sun. The Ulysses spin axis points toward the Earth, varying the angle relative to the Sun. The SWICS ESA steps logarithmically from 0.65 to 59.6 keV/Q in a 13-minute cycle with an instantaneous resolution $\Delta E/E \approx 0.05$ (note that we have simplified $\Delta(E/Q)/(E/Q)$ to $\Delta E/E$ hereafter). The SWICS instruments preferentially cover the solar wind and a large portion of the PUI distributions in the sunward hemisphere in this configuration.

The Cassini Plasma Spectrometer (CAPS) on Cassini made 3-D measurements of the plasma distribution in Saturn’s magnetosphere. CAPS utilizes an ion mass spectrometer (IMS) subsystem to measure ions in the energy/charge range $\sim0.01\text{--}50~\text{keV/q}$ (Young et al. 2004) with energy resolution $\Delta E/E \approx 0.17$. By utilizing a top-hat ESA with large instantaneous FOV ($160^{\circ}\times 8^{\circ}$) and multi-resolution TOF mass spectrometer with mass resolutions $m/\Delta m \approx 70$ and 9, CAPS can measure the distribution functions of multiple species of interstellar PUIs, including $\text{H}^{+}$, $\text{He}^{+}$, and $\text{He}^{2+}$. During Cassini’s travel to Saturn, CAPS provided the first direct observation of interstellar PUIs beyond Jupiter (McComas et al. 2004).

The Charge-Energy-Mass Spectrometer (CHEMS) on Cassini as part of the MIMI neutral/charged particle detection system (Krimigis et al. 2004) measures the energy, charge, and mass of ions in the energy/charge range $\sim3\text{--}220~\text{keV/e}$. Similar to the SULEICA instrument, CHEMS uses a deflection system (ESA), TOF subsystem, and SSD to characterize the energy/charge and mass of ions entering the instrument. CHEMS utilizes a relatively wide FOV ($160^{\circ}\times4^{\circ}$), and during spacecraft rolls, CHEMS can measure three-dimensional particle distributions using its three TOF telescopes. While the primary mission of the CHEMS instrument was to characterize energetic particles in the Saturn magnetosphere, it also provided measurements of energetic particles in the solar wind related to suprathermal PUI tails (Hill et al. 2009).

SOHO is a three-axis stabilized spacecraft devoted to observing the Sun’s atmosphere and particle populations that arrive from the sunward direction. The entrance of the SOHO Charge, ELement, and Isotope Analysis System (CELIAS) CTOF sensor (Hovestadt et al. 1995) continually points into the solar wind direction with a $50^{\circ}\times30^{\circ}$ FOV. It features a relatively large effective entrance aperture ($0.08~\text{cm}^{2}$) to achieve good counting statistics and coverage of the solar wind, including minor species and PUIs. The CTOF ESA subsystem consists of a quadrupole lens at the entrance to a hemispherical $180^{\circ}$ ESA and another lens at its exit into the TOF subsystem. The post-acceleration, TOF, and SSD subsystems are like SWICS, except that the start and stop electrons pass a dual $45^{\circ}$ electrostatic mirror to their respective MCPs. CTOF does not have any angular sectoring, thus providing a 1-D cut through the solar wind and PUI distributions in $E/Q$ in the sunward direction from 0.3 to 34.5 keV/e with logarithmically spaced stepping over 300 sec.

STEREO is a mission with two 3-axis stabilized spacecraft that observe the Sun and solar particles from vantage points drifting at a rate of $22.5^{\circ}$ per year ahead and behind the Earth (Kaiser et al. 2008). The PLasma and SupraThermal Ion Composition (PLASTIC) sensors have a hemispherical top-hat ESA to provide continuously an almost $360^{\circ}\times10^{\circ}$ FOV in the ecliptic plane stepping from 0.3 to 80 keV/Q (Galvin et al. 2008). A $45^{\circ}$ sector, centered on the Sun, has two curved deflector plates upfront, which accept ions from up to $\pm20^{\circ}$ out of the ecliptic plane with a $2\text{--}5^{\circ}$ resolution during each electrostatic angle sweep. The angular resolution in the ecliptic plane is $2\text{--}5^{\circ}$ in the Sun-sector and $22.5^{\circ}$ in the remaining FOV. Exiting the ESA, the incoming ions are post-accelerated by −15 to $-25~\text{kV/Q}$ into PLASTIC’s cylindrical TOF subsystem, which is similar to the TOF subsystem in Cluster CODIF (Reme et al. 1997) and FAST TEAMS (Klumpar et al. 2001). The Sun-sector also contains SSDs to complete the mass and mass/charge analysis of the solar wind composition. With its high angular resolution in both directions in the Sun-sector, PLASTIC was the first sensor to provide a 3-D velocity distribution of PUIs.

### PUI Diagnostics of the Interstellar Gas

#### Interstellar Parameters from the Focusing Cone

Following the interstellar He parameter derivation from the He I 584 Å backscattering observations (Chassefiere et al. 1986; Dalaudier et al. 1984), the early analysis of $\text{He}^{+}$ PUI observations included only the gravitational focusing of the interstellar gas flow. Interstellar neutral He can survive down to a fraction of an au from the Sun. The Sun’s gravity attracts the atoms and bends their trajectories, focusing them into a high-density region of a radially oriented cone shape starting slightly beyond 1 au downstream of the Sun. This region is called the He-focusing cone.

Simulations of the He-focusing cone generally use a “hot” transport model of the interstellar neutral gas (Fahr 1971; Wu and Judge 1979), starting outside the heliosphere with a shifted Maxwellian distribution that assumes a distant bulk flow velocity $\boldsymbol{V}_{\mathrm{ISN}\infty }$ and temperature $T_{\mathrm{ISN}\infty}$. The focusing cone is a substantial density enhancement on the interstellar downwind side of the Sun, which is crossed by the Earth, centered around Dec 4 each year. This location marks the interstellar flow direction in ecliptic longitude $\lambda_{\mathrm{ISN}\infty}$. The flow speed $V_{\mathrm{ISN}\infty}$ and temperature $T_{\mathrm{ISN}\infty}$ determine the cone width $\Delta \lambda _{\mathrm{Cone}}$ approximately as

2 $$ \Delta \lambda _{\mathrm{Cone}} \propto \sqrt{{T_{\mathrm{ISN}\infty}} / {V_{\mathrm{ISN}\infty}^{2}}}. $$

In principle, the PUI flux enhancement integrated over the cone determines the flow speed $V_{\mathrm{ISN}\infty}$ alone via

3 $$ \int _{0}^{2\pi} J_{\mathrm{PUI}} ( \lambda ) d \lambda - J_{\mathrm{PUI}0} \propto \frac{{G M_{\mathrm{S}}} / {r_{\mathrm{O}}^{2}}}{{V_{\mathrm{ISN}\infty}^{2}} / {2}} -1. $$

$J_{\mathrm{PUI}} ( \lambda )$ is the locally measured energy flux density of the PUIs as a function of ecliptic longitude. $J_{\mathrm{PUI}0}$ indicates its base level at longitudes outside the focusing cone. $J_{\mathrm{PUI}} ( \lambda )$ does not vary as strongly with the solar wind speed as the PUI flux (Möbius et al. 1988). $M_{\mathrm{S}}$ is the Sun’s mass, $G$ is the gravitational constant, and $r_{\mathrm{O}}$ is the observer distance from the Sun. The proportionalities in Eqs. (2) and (3) provide insight into physical dependencies without the need to use absolute values. It is evident from Eq. (2) that a cold interstellar gas ($T_{\mathrm{ISN}\infty} = 0$) would lead to a singularity on the downwind side and that the width provides us with the ratio of the thermal to bulk speed of the gas. Equation (3) can be interpreted as the collection of the interstellar gas flow into the cone through a circular impact cross-section around the Sun, whose area is controlled by the ratio of the gravitational potential at the observer distance $r_{\mathrm{O}}$ and the kinetic energy of the interstellar bulk flow at infinity. With the SWICS measurements on ACE, Gloeckler and Geiss (2001a) found that the temperature of interstellar neutral He is about 7500 K, consistent with the direct measurement of the flow vectors of interstellar He gas with Ulysses (Witte et al. 1996). The location of $\text{He}^{+}$ PUI intensity maximum also has allowed Gloeckler et al. (2004) to independently determine the longitude of interstellar flow direction also in agreement with the direct neutral gas measurement on Ulysses (Witte 2004). We note that due to the effects of filtration and scattering (Bzowski et al. 2017; Fraternale et al. 2021; Swaczyna et al. 2021), measurements of interstellar neutrals do not strictly apply to the pristine interstellar medium far from the heliosphere, but rather to the filtered and scattered population entering the heliosphere.

**Fig. 4:**
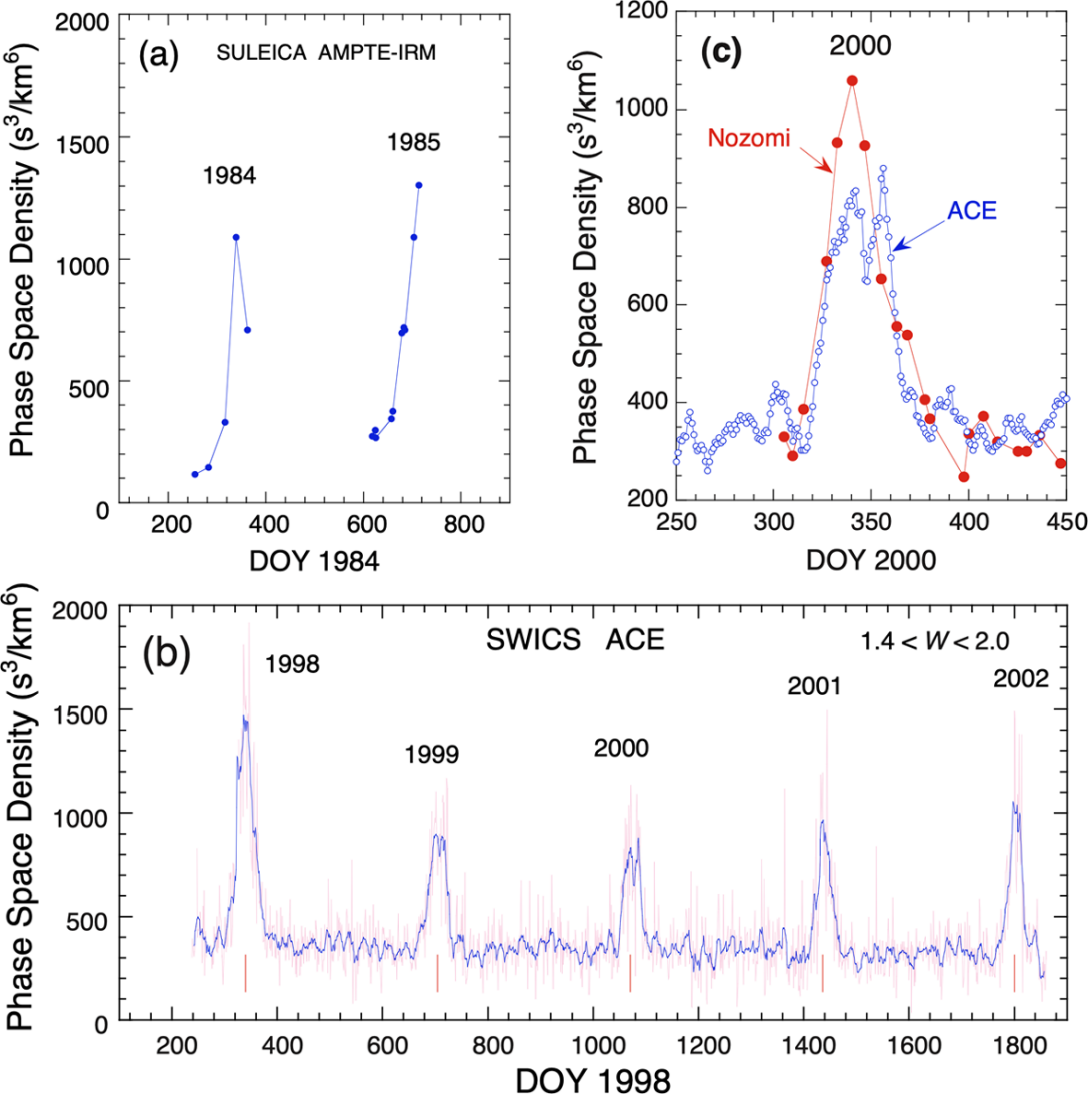
Observations of the He-focusing cone, as seen in the $\text{He}^{+}$ PUI phase space density observed near 1 au in 1984, 1985, and 1998–2002. (**a**) AMPTE-IRM SULEICA and (**b**) ACE SWICS made observations near Earth and L1, respectively. (**c**) ACE and Nozomi observations are also compared for observations in 2000. The peak location of the He focusing cone is found on DOY 339.75, corresponding to Earth’s crossing of the cone center at longitude $\lambda = 74.6^{\circ}$. From Gloeckler et al. (2004). © ESO. Reproduced with permission

However, ionization depletes the density flowing through the heliosphere, thus reducing the density in the focusing cone for an increasing total ionization rate. Conversely, increased ionization also increases the production of PUIs, but on a different spatial scale than the depletion of the interstellar gas. Therefore, a careful review and determination of the ionization rates based on solar UV and solar wind observations must accompany the PUI analysis (McMullin et al. 2004; Ruciński et al. 1996). The first quantitative determination of the interstellar parameters from PUI observations (Möbius et al. 1995) contained all these ingredients but concluded with substantial uncertainties. The influence of the ionization rates on the density of the focusing cone was immediately evident in the display of continuous PUI observations from two solar activity cycles, as shown in Fig. 4 with AMPTE-IRM SULEICA, ACE SWICS, and NOZOMI observations (Gloeckler et al. 2004). In addition, the PUI phase space density used in this representation showed strong day-to-day variations. A 9-day sliding average reduced them significantly, but the focusing cone always appeared to contain sub-structures. These variations that have multiple sources posed substantial challenges for the PUI diagnostics of interstellar parameters.

Early on, it was apparent that the IMF orientation played a substantial role in forming the PUI velocity distribution and the energy flux densities derived from them. The more the IMF deviates from an orientation perpendicular to the solar wind, the lower the PUI density near the PUI cut-off (Gloeckler et al. 1995; Möbius et al. 1998b). In an attempt to work around this challenge, Möbius et al. (1995) selected time intervals with near perpendicular IMF for their analysis, thus substantially reducing their database. The use of anisotropic PUI distributions (Gloeckler et al. 1995) and so-called hemispherical distributions with restricted pitch-angle diffusion between the sunward and anti-sunward hemisphere (Isenberg 1997) partially addressed this challenge. In fact, it is not only the IMF orientation that shapes the PUI distribution. The assumption that scattering of PUIs into a shell in velocity space in the SW frame is very fast compared with the time scale of adiabatic cooling is often not valid. The scattering rate depends on the power of Alfvénic fluctuations in the resonant frequency range for the PUIs, as demonstrated in observations with SOHO CTOF and CELIAS (Saul et al. 2007, 2004a,b). During time intervals with almost radial magnetic field, the observed PUI distributions become increasingly isotropic as the wave power in the resonant regime grows stronger. For $\text{He}^{+}$, these waves are almost exclusively intrinsic fluctuations in the solar wind.

**Fig. 5:**
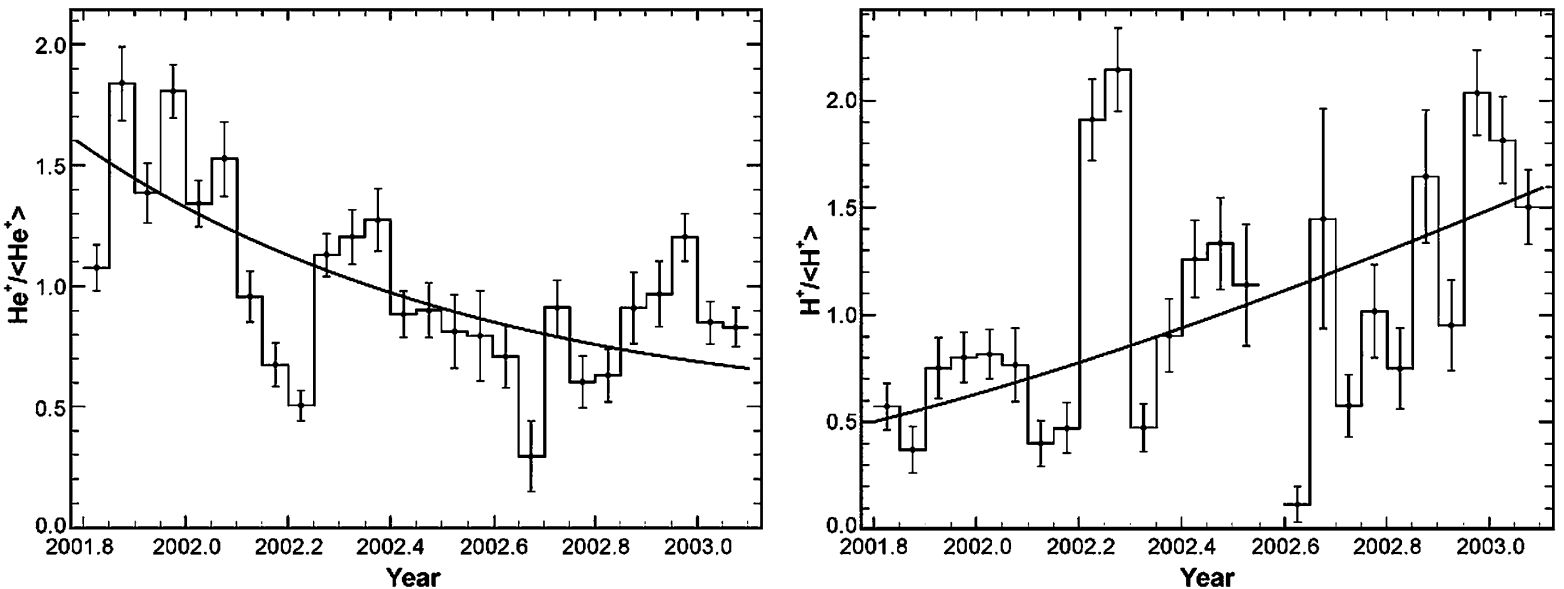
Cassini CAPS measurements of $\text{He}^{+}$ (left) and $\text{H}^{+}$ (right) PUI fluxes divided by their 17-month average as Cassini traveled between 6.4 and 8.2 au downstream of the Sun. As demonstrated by the model results (solid curves), the $\text{He}^{+}$ PUI density decreased over long-term as Cassini traveled away from the interstellar He-focusing cone, and the $\text{H}^{+}$ PUI density increased over long-term as it traveled outside of the interstellar H shadow. From McComas et al. (2004)

However, other effects also contribute strongly to the observed temporal variations of the PUI fluxes. Solar wind compressions and rarefactions also modulate the PUI distributions, which appear to be tied to the solar wind, as first noticed by a curious correlation between $\text{H}^{+}$ and $\text{He}^{+}$ PUI densities despite their starkly different ionization histories (Gloeckler et al. 1994b). Following related compressions and rarefactions through the focusing cone with the two STEREO spacecraft, it became evident that solar wind compressions enhance the PUI densities, thus imparting a comparably strong sub-structure on the annual variation across the focusing cone (Möbius et al. 2010). Cassini CAPS measurements also showed significant variability in interstellar $\text{H}^{+}$ and $\text{He}^{+}$ PUI intensity as Cassini traveled through the “interstellar H shadow” between $\sim6.4$ and 8.2 au downwind of the Sun (McComas et al. 2004). While a PUI model utilizing LISM parameters consistent with SWICS measurements reproduced the long-term evolution of the interstellar $\text{H}^{+}$ and $\text{He}^{+}$ PUI densities (Fig. 5), the underlying, short-term variability observed by CAPS was not reproduced by the steady-state model, and may have originated from PUI enhancements in compression regions (McComas et al. 2004).

In addition, compressions and rarefactions modulate the entire PUI distribution function. Compressions steepen the falloff from the solar wind to the cut-off, and the expanding solar wind in rarefaction regions flattens it, which also leads to a variation of the PUI cooling in the solar wind over the solar cycle (Chen et al. 2013). Chen et al. (2015) demonstrated the efficacy of these compressions and expansions in a simulation. Furthermore, solar wind compressions shift the PUI cut-off to higher speeds, acting almost like an adiabatic compression on the PUI velocity distribution (Bower et al. 2019; Saul et al. 2003).

The aforementioned effects on the PUIs could not explain all the observed PUI variations, which fed the suspicion that PUI transport effects may be at play. Adiabatic focusing of the PUIs in the divergent IMF and drifting of PUIs along the IMF with different speeds according to their pitch-angles may mix distributions from different source regions. These processes widen and shift the focusing cone in ecliptic longitude, depending on the IMF orientation (Möbius et al. 1996). Assuming various diffusion coefficients, Chalov and Fahr (2006, 1999) found a deviation of the PUI cone center from its neutral gas source by up to $5^{\circ}$ in longitude. All these approaches still invoke symmetries and effective diffusion in velocity space.

However, when studied with high angular and energy resolution, PUI distributions show a substantial imprint of the original ring distribution that arises from the injection of the ions into the IMF orientation at their source location (Drews et al. 2015; Oka et al. 2002). Using a kinetic trajectory approach in a realistic IMF with embedded fluctuations (Schwadron et al. 2010b), Quinn et al. (2016) found a shift from $0.36^{\circ}$ to $1.8^{\circ}$, depending on which effects they included. Using just pitch-angle diffusion returns the lowest value, adding adiabatic focusing adds $1.25^{\circ}$, with perpendicular diffusion, and particle drift adding minor contributions.

#### Interstellar Parameters from the PUI Shell Cut-off Velocity

In addition to identifying the focusing cone for interstellar Ne, Drews et al. (2012) found a distinct maximum in the PUI flux in the upwind direction for He, O, and Ne, with a much smaller variation in longitude than for the focusing cone, which they referred to as the PUI crescent. They interpreted this longitudinal flux variation as an increasing depletion of the interstellar neutral gas from the upwind direction toward the downwind side, overcompensated by the gravitational focusing for He and Ne. However, Sokół et al. (2016) disputed this interpretation with detailed simulations of the interstellar gas depletion based on the observed ionization rates in the heliosphere. Instead, the PUI cut-off speed variation with the radial component of the interstellar gas flow velocity $v_{r}$ with ecliptic longitude at 1 au causes a modulation of the PUI velocity distribution in and out of the sensor energy bin that straddles the cut-off similar to the observed flux variation. The difference of the PUI cut-off speed due to the interstellar gas flow speed of $\approx50~\text{km}\,\text{s}^{-1}$ at 1 au had been evident in a comparison between the observations with AMPTE-IRM SULEICA on the downwind side and with SOHO CELIAS CTOF on the upwind side of the gas flow (Möbius et al. 2001).

**Fig. 6:**
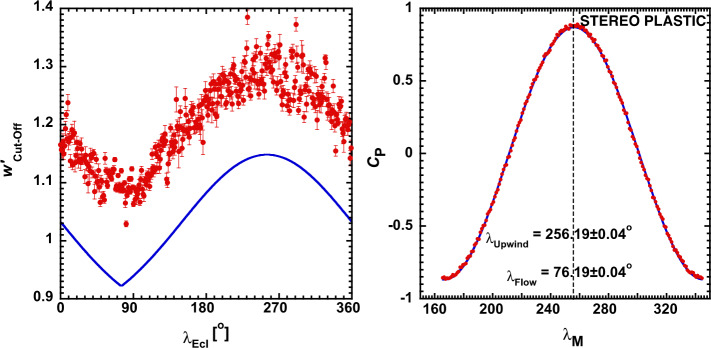
(Left) $w'_{\text{Cut-off}}$ in the solar wind frame obtained from a fit to each daily PUI distribution with the statistical fit errors. The model curve shows a constant offset, likely due to the simplifications that do not consider the exact cut-off shape nor an integration over the sensor FOV and energy bands. (Right) Pearson correlation coefficient between the cut-off values on the left and the same values mirror-imaged about a mirror line shown as a function of $\lambda_{M}$. Also shown is a fit with a cosine function. The maximum correlation is associated with the upwind direction of the ISN flow. From Möbius et al. (2015, 2016b). © AAS. Reproduced with permission

As demonstrated by Möbius et al. (2015), the symmetric variation of $v_{r}$ or the cut-off speed $w_{\text{cut-off}}$ with the ecliptic longitude $\lambda _{\mathrm{ecl}}$ relative to the interstellar gas flow direction $\lambda_{\mathrm{ISN}\infty}$ outside the heliosphere enables a precise determination of this direction. It is complementary to the 4-dimensional interstellar gas parameter tube, found with direct neutral gas imaging by IBEX-Lo (McComas et al. 2012; Möbius et al. 2012; Schwadron et al. 2015). Figure 6 shows the cut-off speed $w_{\text{cut-off}} ' = {v_{\text{cut-off}} '} / {V_{\mathrm{SW}}}$, as obtained with STEREO PLASTIC over seven years, in the solar wind frame on the left. The high angular and energy resolution of PLASTIC enabled this transformation, which makes the cut-off value largely independent of the IMF direction. The solid blue line shows a simplistic model for $w'_{\text{cut-off}}$, using $v_{r}$ according to Eq. (5) in Möbius et al. (2015), not accounting for the exact shape of the distribution function and integration over the PLASTIC energy and angle bins, hence the constant offset. The right side of Fig. 6 shows the Pearson correlation coefficient for correlating the function on the left with its mirror image about $\lambda _{\mathrm{M}}$. The maximum as a function of $\lambda _{\mathrm{M}}$ indicates mirroring about $\lambda_{\mathrm{ISN}\infty}$. The small error bars cited in the result by Möbius et al. (2016b) solely contained the statistical fit errors, needing further detailed analysis of other stochastic and systematic error sources. Taut et al. (2018) performed a detailed analysis of how the stochastically distributed solar wind disturbances over consecutive years influenced the result, using a method by Drews et al. (2012), and culled the data for distortion of the cut-off by PUI acceleration using $\text{He}^{2+}$ distributions to detect these events. Bower et al. (2019) studied how solar wind compressions and rarefactions moved the cut-off speed, energizing or de-energizing the PUI distribution. They developed a criterion to cull data for these cut-off changes that are unrelated to the interstellar flow. Both analyses returned a consistent interstellar flow direction in ecliptic longitude of $\lambda_{\mathrm{ISN}\infty} = 75.6\pm0.5^{\circ}$. In addition, they provided valuable information on the PUI dynamics in response to variations in the solar wind.

### Observations of Interstellar PUIs with SWICS

The SWICS instrument on Ulysses revolutionized the measurement of PUIs. The main objective of the NASA-ESA joint Ulysses mission was to explore the heliosphere at high latitudes, over the Sun’s poles. It covers heliographic latitudes from the equator to $80^{\circ}$ both North and South and a radial distance range between 1.3 and 5.4 au from the Sun. Ulysses SWICS measurements provided a detailed look at PUI distributions, their composition, and the ubiquity of suprathermal tails in the solar wind.

#### Measurements of Interstellar PUI Spectra and Composition

**Fig. 7:**
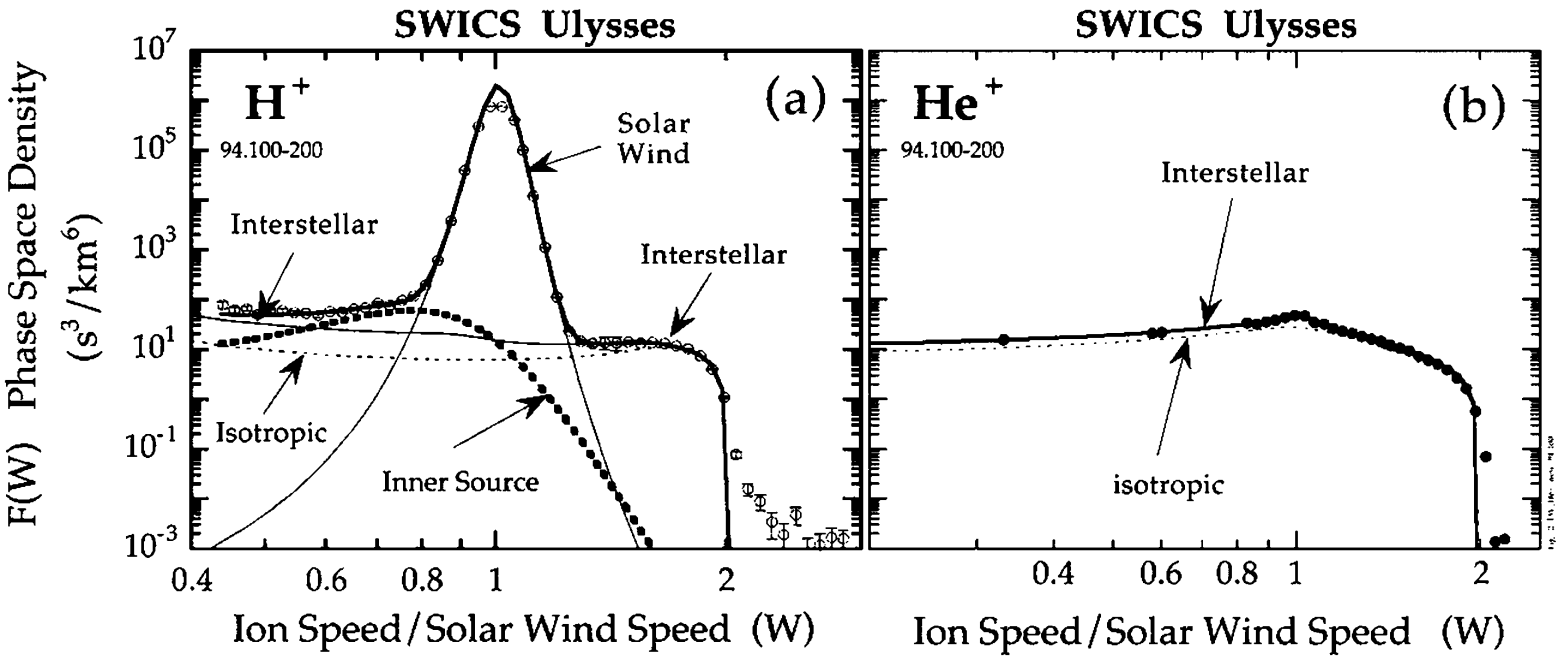
Velocity distribution functions as a function of $w = v/V_{\mathrm{SW}}$ for $\text{H}^{+}$ and $\text{He}^{+}$ observed by Ulysses SWICS during a time when Ulysses was in the high latitude, fast solar wind. Multiple populations of ions were observed, including the core SWIs, interstellar PUIs, and the inner source PUIs. Fits to the interstellar PUI distributions assuming strong pitch angle scattering (“isotropic”, dashed) and weak pitch angle scattering (solid) are also shown, indicating better agreement under the assumption of weaker scattering allowing anisotropic distributions to form. From Gloeckler and Geiss (2001b). © Springer. Reproduced with permission

Gloeckler et al. (1993) reported the detection of interstellar $\text{H}^{+}$ PUIs with the Ulysses SWICS instrument (Gloeckler et al. 1992) at a distance of 3–4.8 au from the Sun, i.e., at the edge of the heliospheric H ionization cavity. The interstellar PUI distribution functions are uniquely determined by their sharp cutoffs at approximately $2 V_{\mathrm{SW}}$ (see Figs. 7 and 12). Ulysses SWICS also observed $\text{He}^{2+}$ PUIs which allowed it to directly derive the absolute interstellar He density, as well as $\text{N}^{+}$, $\text{O}^{+}$ and $\text{Ne}^{+}$ PUIs at reduced levels due to the scarcity of their parent neutral populations in the local interstellar medium (Gloeckler and Geiss 2001b, 1998).

Figure 7, from Gloeckler and Geiss (2001b), shows the velocity distribution function of $\text{H}^{+}$ and $\text{He}^{+}$ as measured by the SWICS on Ulysses in the spacecraft frame of reference. The $\text{H}^{+}$ spectrum contains both PUIs and SWIs. Between $w = \sim 0.8$ and $\sim1.3$ are solar wind $\text{H}^{+}$. Interstellar $\text{H}^{+}$ PUIs are above $w = \sim1.3$ or below $w = \sim 0.6$. The $\text{He}^{+}$ spectrum contains entirely interstellar PUIs. The most prominent feature of the spectra is the sharp drop-off at $w\cong2$, which is the expected cutoff speed of freshly born PUIs in the spacecraft reference frame. The spectral shapes of $\text{H}^{+}$ and $\text{He}^{+}$ PUIs are different. The distribution of $\text{H}^{+}$ PUIs is more flattened than that of $\text{He}^{+}$. This flat spectrum is because PUIs produced earlier at smaller radial distances have been adiabatically cooled to a lower (internal) thermal speed while propagating out with the solar wind. The density of interstellar neutral H is depleted in the inner heliosphere by ionization processes, and thus interstellar neutral He is the dominant neutral species closer to the Sun because of its high ionization potential. In effect, this means that there are more $\text{He}^{+}$ PUIs than $\text{H}^{+}$ PUIs close to the Sun. As discussed in Sect. 2.1, after adiabatic cooling, PUIs appear at low thermal speeds relative to the solar wind with high intensity (see Fig. 1). This explains why the $\text{He}^{+}$ PUI spectrum decreases from the solar wind speed $w = 1$. The spectral shape of the PUIs can be used to deduce not only the ionization rate but also the density of the parent atoms in the heliosphere.

To understand the measured PUI spectra shown in Fig. 7, fits with consideration of PUI production and cooling history, instrument geometry, and particle detection efficiency must be performed. Details of the fits can be found in Gloeckler et al. (1995). Using the distribution function derived by Vasyliunas and Siscoe (1976), shown in Eq. (1), and an assumption of interstellar neutral density given by Thomas (1978), Gloeckler et al. (1995) found that it is impossible to fit the observations with an isotropic distribution for $\text{H}^{+}$ PUIs. Instead, the $\text{H}^{+}$ PUIs must have a sizable sunward-to-antisunward intensity ratio of $\sim3$ to 1. If the anisotropy is a result of diffusion, a radial mean free path of $\sim2~\text{au}$ was derived in the analysis by Gloeckler et al. (1995). Based on the theory that particles tend to have difficulty getting scattered across the $90^{\circ}$ pitch angle due to the lack of plasma turbulence power at small wavelengths (Earl 1974; Jones et al. 1978), Schwadron et al. (1996) interpreted the sunward-antisunward anisotropy seen by Gloeckler et al. (1995) as a bi-hemispherical pitch-angle distribution. The large radial mean free path of 2 au can be translated into a much larger parallel mean free path ($>10~\text{au}$) given the spiral angle of the IMF at the spacecraft where the measurements are made. This result tells us that $\text{H}^{+}$ PUIs are almost scatter-free at this distance from the Sun. $\text{He}^{+}$ PUIs are also best fit with an anisotropic distribution (Fig. 7b) (Gloeckler and Geiss 2001b), although the disparity between the sunward and antisunward distributions is less significant than for $\text{H}^{+}$. This may be partly due to the larger amount of time that $\text{He}^{+}$ PUIs have to isotropize while advecting with the solar wind out to this distance, since the ionization cavity size for interstellar He is $\sim8$ times smaller than for interstellar H (Sokół et al. 2019b).

#### Origin of PUI Pitch Angle Scattering in the Solar Wind

We note that the measurements shown in Fig. 7 were taken whilst Ulysses was in a high-speed SW stream at high latitudes, where turbulence levels are typically lower than in the slower SW (Gloeckler and Geiss 1998). During these observation times, the anisotropy of PUIs was apparent, where a significant component of PUIs are streaming radially-inward in the solar wind frame (Gloeckler et al. 1995). On the other hand, observations of PUI distributions in slower SW at low latitudes showed little evidence of anisotropy (Gloeckler and Geiss 2001a). These measurements suggests that PUI pitch angle scattering, at least at these distances from the Sun, is primarily driven by intrinsic magnetic fluctuations in the solar wind.

The anisotropy of $\text{H}^{+}$ and $\text{He}^{+}$ PUI distributions as observed by Ulysses SWICS leads to the question: what is the origin of fluctuations responsible for pitch-angle scattering PUIs as a function time, SW speed, and distance from the Sun? As mentioned above, observations of higher PUI anisotropies in faster SW streamlines suggests that magnetic fluctuations intrinsic to the solar wind are responsible for their evolution in pitch angle, at least within a few au of the Sun. There are clear correlations between magnetic wave power near the ion cyclotron resonance frequency and the scattering rate of PUIs in the solar wind (Saul et al. 2007, 2004a,b). Magnetic fluctuations created by the isotropization of unstable PUI beams should also be present near the ion cyclotron resonance frequency, but direct measurements of the creation and evolution of PUI-generated waves are very difficult to make. Nevertheless, numerous studies have searched for the signatures of PUI-generated waves at the expected frequencies. Extensive analyses of Ulysses magnetic field observations by Cannon et al. (2014a, 2014b) found that while there were clear signatures of PUI-generated waves, they were much more scarce than anticipated, where signatures appeared correlated with times of low background turbulence in the solar wind. Fisher et al. (2016) found similar signatures of PUI-generated waves using ACE/MAG observations, with a similar conclusion for the scarcity of the measurement (see also Smith et al. 2017). Hollick et al. (2018a, 2018b, 2018c) extended analyses of PUI-generated waves to $\sim43~\text{au}$ from the Sun with Voyager observations, where $\text{H}^{+}$ PUIs dominate the thermal pressure of the solar wind (McComas et al. 2017b). Hollick et al. not only found PUI-generated waves with properties consistent with previous analyses, but also found the unique presence of right-hand polarized waves that appeared to be correlated with times when the IMF had a significant non-radial component. All these analyses thus far observed similar signatures of enhanced wave power near the PUI cyclotron gyrofrequency, waves with predominantly left-hand polarization as expected from theory (except possibly within non-radial IMF), and that the wave signatures largely appeared only when the background turbulence was low.

The analyses summarized here provide an extensive list of evidence for PUI-generated waves from 1 au to $\sim43~\text{au}$; however, we note that the studies’ authors have described in detail the potential uncertainties of their analyses. It is not yet completely clear what the primary driving force is for PUI pitch angle scattering in different SW conditions, but current evidence shows clear correlations with fast vs slow SW, background turbulence levels, and rarefaction regions.

#### Presence of Inner Source PUIs

In addition to interstellar PUIs that are continuously produced above certain radial distances in the heliosphere before their interstellar neutral sources are completely ionized by the solar radiation, solar wind electron impact, and charge exchange, the SWICS instrument discovered a new source of PUIs near the Sun. Most noticeable are $\text{C}^{+}$ ions (Geiss et al. 1995), which are clearly distinguishable from interstellar PUIs by their different velocity distribution. All interstellar PUIs feature a distribution with a very sharp cut-off near $2V_{\mathrm{sw}}$ in the observer frame, i.e., where freshly ionized PUIs are injected into the solar wind close to the observing spacecraft. The thermal speed of inner source ions is so low due to adiabatic cooling, that they are almost comparable to that of the SWIs. However, given their low ionization potentials compared to the temperature of the solar corona, it is unlikely that they originated in the corona. Instead, they are most likely PUIs produced within a fraction of an au of the Sun.

The presence of inner source PUIs (Geiss et al. 1995; Gloeckler et al. 2000a; Gloeckler and Geiss 2001b, 1998) indicates that there are neutral sources of these elements. Atomic or molecular forms of these elements cannot survive in the environment for long. Most likely, they arise from the solar wind interaction with dust grains, possibly as sputtered products (Allegrini et al. 2005; Gloeckler et al. 2000a; Gruntman 1996; Schwadron et al. 2000; Wimmer-Schweingruber and Bochsler 2003) that are picked up by the solar wind close to the Sun, thus evolving into a distribution concentrated around the core SWIs.

On the other hand, inner source PUIs provide insight into the dust population in the inner heliosphere and their interaction with the solar wind. Several possible ways that inner source PUIs to be produced from interstellar dust interactions with the solar wind have been proposed. Most proposed mechanisms involve charge exchange, neutralization, and reionization of solar wind ions as they pass through, nearby, or scatter off of dust grains (Quinn et al. 2018; Schwadron et al. 2000; Wimmer-Schweingruber and Bochsler 2003).

Recent detections of dust impacts by the FIELDS instrument onboard Parker Solar Probe (Fox et al. 2016) provided closer measurements of dust to the Sun than any spacecraft before it (down to $\sim0.1~\text{au}$). Szalay et al. (2021) determined that the cross section derived from sub-micron sized dust grains is not sufficient to account for the inner source PUI production rate. Rather, they propose that dust grains with radii $<50~\text{nm}$, which are susceptible to trapping near the Sun by electromagnetic forces, may be the source of these PUIs.

#### Putting Interstellar Gas Measurement Techniques into Perspective

In an attempt to consolidate the determination of the interstellar gas parameters for He, an ISSI science team put three available in-situ observation methods for the interstellar gas inside the heliosphere, i.e., UV backscattering, PUI, and neutral atom imaging observations, into perspective (Möbius et al. 2004). In a nutshell, PUI diagnostics provide an excellent local diagnostic tool for the interstellar neutral atom distribution. They enable precise measurements of the interstellar flow direction in ecliptic longitude (Bower et al. 2019; Möbius et al. 2015; Taut et al. 2018), the interstellar He density, independent of the absolute calibration of the sensors (Gloeckler et al. 1997; Gloeckler and Geiss 1998), and abundance ratios for most of the elements with high ionization potential (Gloeckler and Geiss 2001a). The SWICS detection of $\text{He}^{2+}$ PUIs specifically, as predicted by Ratkiewicz et al. (1990) and Ruciński et al. (1996), enabled the determination of the interstellar He density based on a measurement of the $\text{He}^{2+}$ PUI to SWI ratio with the same sensor, i.e., using its substantially more precise relative calibration of a single ion over energy in the analysis. The uncertainty of the result, $n_{\text{He}} = 0.0153\pm 0.0018~\text{cm}^{-3}$ (Gloeckler et al. 1997; Gloeckler 1996; Gloeckler and Geiss 1998), mostly rests on the knowledge of the He to $\text{He}^{2+}$ charge exchange cross-section (Barnett et al. 1990; Ruciński et al. 1998) and counting statistics. The comparison between the composition obtained from PUI analysis and the composition in the local interstellar cloud (Frisch and Slavin 1996; Slavin and Frisch 2002) suggests that the composition of interstellar H, O, and N are affected by filtration by hot plasmas in the heliosheath, but not so for the noble gases He and Ne.

However, deducing the interstellar gas speed relative to the Sun, temperature, and latitudinal flow direction still depends on the focusing cone. Their determination is fraught with interference from PUI transport effects (Gloeckler et al. 2004, 1994b; Möbius et al. 1998b, 1995) and variations of the local PUI density due to solar wind compression and rarefaction regions (Chen et al. 2013; Möbius et al. 2010).

Conversely, direct interstellar neutral gas imaging provides access to the entire dynamic and kinetic state of the neutral gas for He with Ulysses GAS (Bzowski et al. 2014; Witte 2004; Witte et al. 1996; Wood et al. 2015) and with IBEX (Bzowski et al. 2012; Möbius et al. 2012, 2009a,b; Swaczyna et al. 2018). IBEX has expanded this capability to include other species: O (Bochsler et al. 2012; Möbius et al. 2009a,b; Schwadron et al. 2016), Ne (Bochsler et al. 2012; Park et al. 2014), and H (Saul et al. 2012; Schwadron et al. 2013). IBEX measurements available for species that can turn into a stable negative ion, such as H and O, or are unambiguously identifiable through their sputtering characteristics (Bochsler et al. 2012; Möbius et al. 2012), limiting the range of interstellar species compared to PUI diagnostics.

The observation of backscattered solar UV, which enabled the detection of interstellar gas inside the solar system for H (Bertaux and Blamont 1971; Thomas and Krassa 1971) and He (Paresce et al. 1973; Weller and Meier 1974), is limited to just these two species. Obtaining the dynamic parameters and interstellar H and He densities is highly dependent on concurrent solar radiation and ionization rate measurements. However, the backscatter observations provide the longest data records of the surrounding interstellar gas.

#### Suprathermal Tails in the Solar Wind

**Fig. 8:**
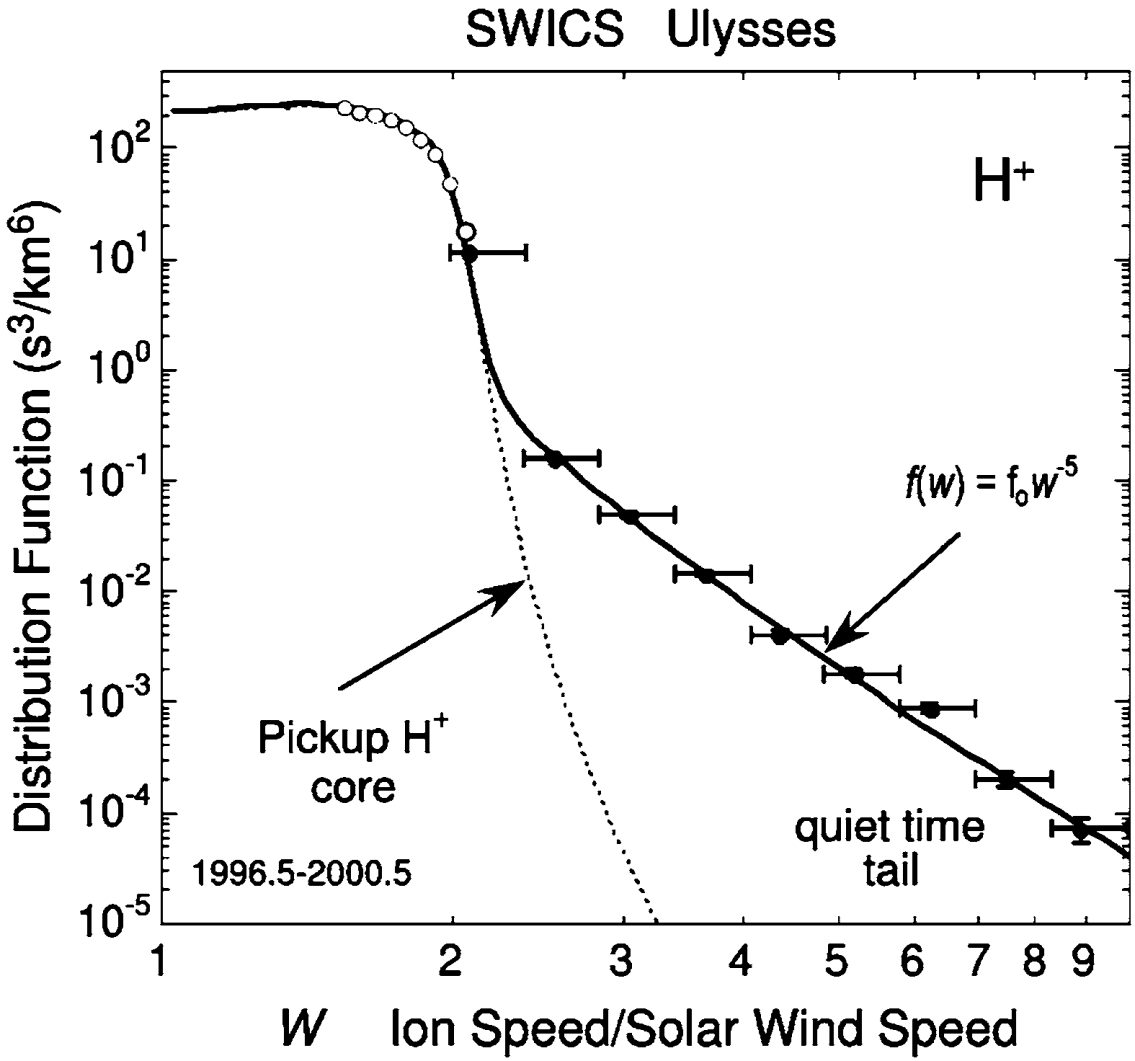
$\text{H}^{+}$ PUI distribution function as measured by Ulysses SWICS. Measurements were taken from 1996–2000 at $\sim4.8~\text{au}$ from the Sun. Model fits to the PUI core (dotted curve) and suprathermal tail with slope $w^{-5}$ yield a total distribution fit (solid curve). From Fisk and Gloeckler (2006). © AAS. Reproduced with permission

**Fig. 9:**
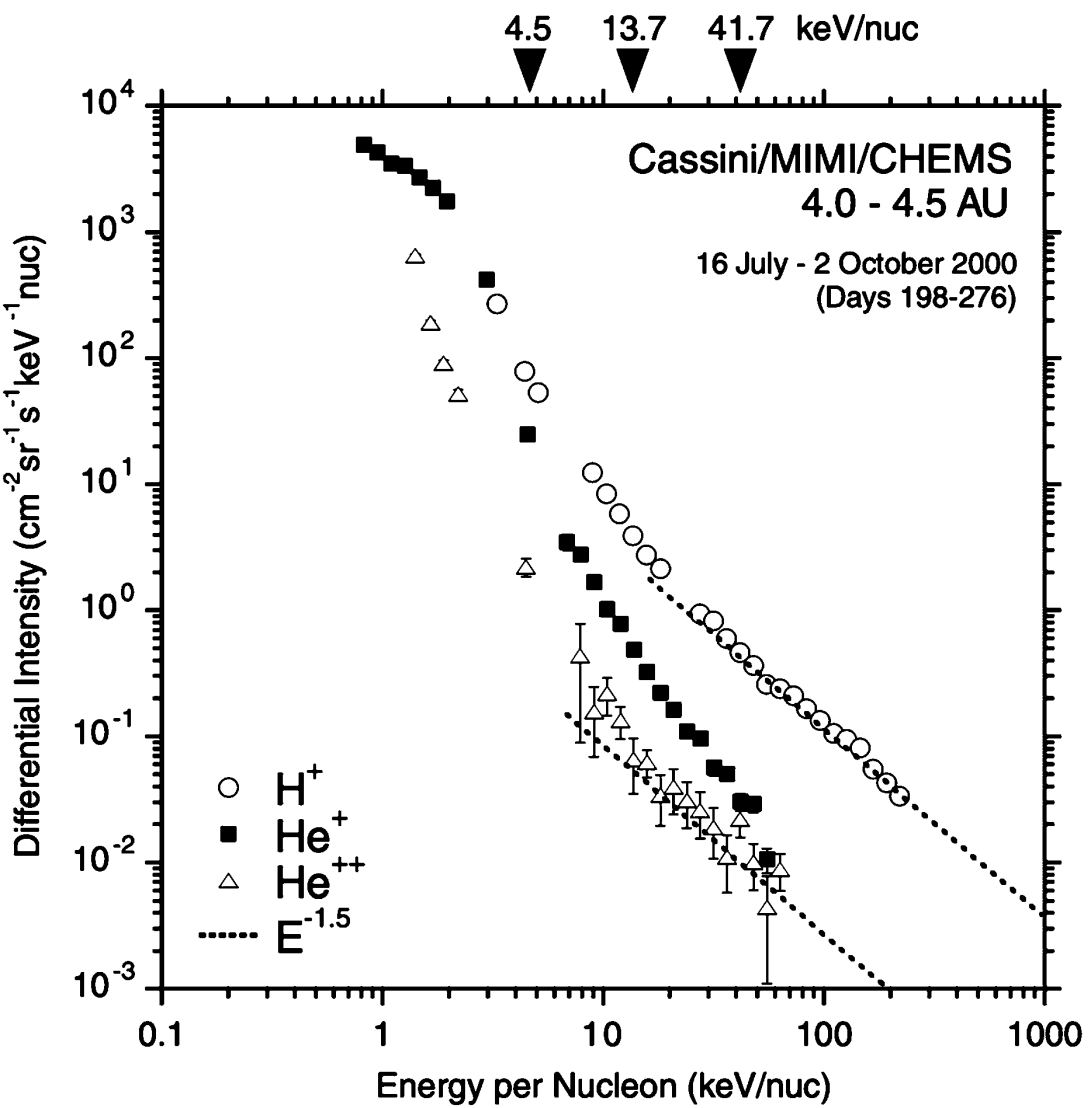
Observations of $\text{H}^{+}$ and $\text{He}^{+}$ PUIs and solar wind $\text{He}^{2+}$ from Cassini CHEMS measured during quiet times in the solar wind. The $\text{E}^{-1.5}$ common spectrum proposed by Fisk and Gloeckler is also shown. From Hill et al. (2009). © AAS. Reproduced with permission

Gloeckler et al. (2000b) first pointed out that suprathermal tails, preferentially of PUIs, occur in the solar wind even during quiet times. They defined such quiet times either by selecting for very slow solar wind ($V_{\mathrm{sw}}\leq 320~\text{km/s}$) (Gloeckler et al. 2008, 2000b) or for a low upper limit in the observed particle rate (Fisk and Gloeckler 2006). They stressed that these quiet time tails exhibit a common spectrum with a $v^{-5}$ power law in the solar wind frame (see Fig. 8), which turns into an exponential rollover at higher energies (Gloeckler et al. 2008). Other spacecraft observations revealed similar measurements. Analyses of Cassini CHEMS (Krimigis et al. 2004) observations of $\text{H}^{+}$, $\text{He}^{+}$, and $\text{He}^{2+}$ suprathermal tails showed the presence of $v^{-5}$ power law tails both during quiet time periods (Hill et al. 2009), e.g., Fig. 9, as well as during active periods (Hill and Hamilton 2010) in the solar wind between $\sim5$ and 9 au from the Sun. New Horizons PEPSSI measurements of suprathermal $\text{He}^{+}$ showed spectral slopes between $v^{-4}$ and $v^{-6}$, which varied with the solar cycle and showed correlations with high-speed solar wind streams (Kollmann et al. 2019). In contrast, other studies of event samples for $\text{He}^{+}$ (Popecki et al. 2013) and extensive surveys for a variety of species indicated substantial variations in the spectral indices, preferentially toward softer spectra (Dayeh et al. 2017; Desai et al. 2006).

Claiming the ubiquity of the $v^{-5}$ spectrum, Fisk and Gloeckler (2014, 2008, 2006) proposed an acceleration model for quiet times in the solar wind that invokes compressional turbulence and redistributes the particle energies analogous to a thermodynamic steady state. However, Jokipii and Lee (2010) pointed to some weaknesses in the model derivation. Alternatively, stochastic acceleration by waves can produce spectra close to $v^{-5}$ with adiabatic cooling included (Zhang 2010; Zhang and Lee 2013). Reconnection in stochastically distributed magnetic islands leads to a power law that approaches $v^{-5}$ in the outer heliosphere, preferably downstream of shocks (Drake et al. 2013; Zank et al. 2014; Zhao et al. 2018). Schwadron et al. (1996) invoked transit-time damping in compression regions, which could also work in quiet solar wind albeit at reduced rates. While not directly quantifying the existence of $v^{-5}$ suprathermal tails, Schwadron et al. (1999) revealed Ulysses SWICS measurements of $\text{H}^{+}$ and $\text{He}^{+}$ PUIs with significant enhancements in high-latitude compression regions. They demonstrated that PUI compression with a large scattering mean free path both inside and outside the compression regions is consistent with the observations. All alternative models appear to be most effective in and near solar wind compressions and thus would require transport of the tails into quiet regions. Adopting such a scenario, any models that accelerate particles in CIRs and CMEs (Fisk and Lee 1980; Giacalone et al. 2002; Richardson 2004) could work. In a synergistic approach, Schwadron et al. (2010a) showed that a stochastic superposition of power law, exponential, and Gaussian spectra would naturally combine into a near $v^{-5}$ power law. The emergence of power law tails with slopes near $v^{-5}$ are also a natural occurrence of space plasma distributions in stationary states far from equilibrium (Livadiotis and McComas 2010, 2009).

Whether a genuine quiet time acceleration is necessary or whether tails from compressions or shocks could migrate into quiet regions required a systematic study of spatial variations of the tails around compression regions and their potential dependence on compression and shock strength. Studying the immediate vicinity of coherent structures and shocks, Tessein et al. (2013) found that the suprathermal ion fluxes peak at the center of the structures and increase with the strength of associated magnetic discontinuities.

**Fig. 10:**
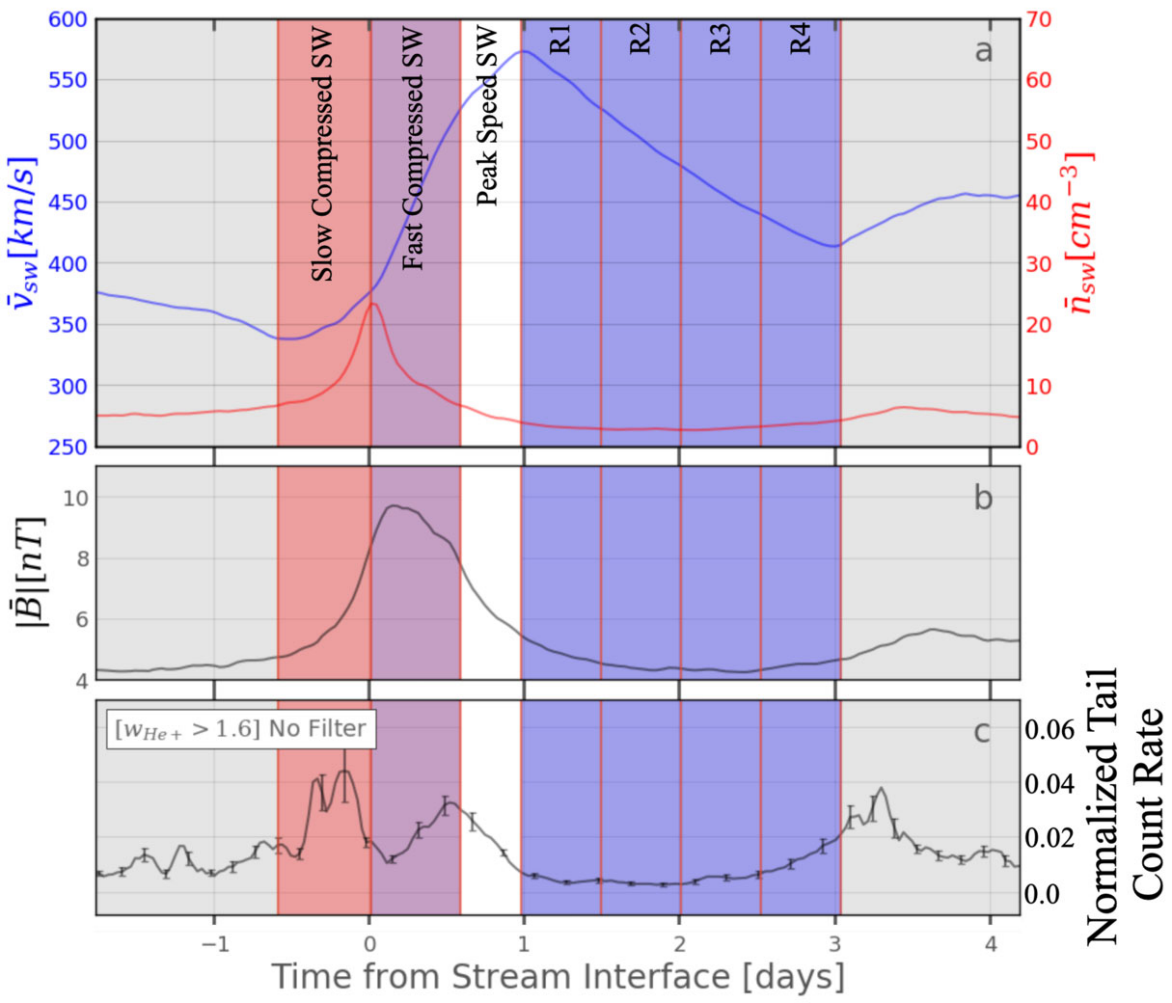
Temporal evolution of solar wind speed and density (top), magnetic field strength (center), and suprathermal tail count rate, normalized to the PUI count rate (bottom) across a solar wind compression region in a superposed epoch analysis. Adapted from Möbius et al. (2019)

A superposed epoch analysis of the PUI response to solar wind compressions in connection with the PUI cut-off variation discussed above (Bower et al. 2019) presented the opportunity to study the variation of the tails with distance from the compression or shock as the data already included the $\text{He}^{+}$ tails in the appropriate form. The superposed epoch analysis also addressed the challenge that tails in quiet time regions show low counting statistics. Generally, tails appeared most intense in the center of the compression regions and weakest in the center of the rarefaction region, as can be seen in Fig. 10, for the tail count rate, normalized by the PUI count rate which is presumably the source for the tails. The spectra softened in stronger compressions and from the slow compressed to the fast compressed solar wind. In the slow compressed wind, they are closest to $v^{-5}$ (Möbius et al. 2019). The very slow solar wind, used in one of the quiet time criteria (Gloeckler et al. 2000b), occurs just before the following compression region, with the slow compressed wind up front. A follow-on study of shocks demonstrated that the power-law index varies with the shock compression ratio as predicted by diffusive shock acceleration. Also, the weighted mean index for all observed shocks is close to −5 (Bower et al. 2021), which supports the finding by Schwadron et al. (2010a). Comparing these results with a model that includes the appropriate acceleration at the compression or shock and the transport into the rarefaction region, which is mainly quiet, will be the next logical step to test the alternate scenarios for these tails.

#### PUIs as an Effective Source Distribution for Acceleration

ACRs featured compositional signatures of the interstellar neutral gas entering the heliosphere. This finding led to ACR models involving the acceleration of ions of interstellar origin (Fisk et al. 1974; Klecker 1977; Pesses et al. 1981). These models suggested that ions generated from neutral atoms may be accelerated more effectively than the bulk solar wind. The discovery of interstellar PUIs fostered this connection (Hovestadt et al. 1985; Möbius et al. 1985b) and allowed further study of the efficacy of PUI injection into acceleration processes in the solar wind.

Although unnoticed at the time, the first observational indication for the very effective injection of PUIs into an acceleration process in the inner heliosphere may have been the detection of widespread and unusually high $\text{He}^{+}/\text{He}^{2+}$ ratios in energetic interplanetary particles (Hovestadt et al. 1984). Gloeckler et al. (1994a) first showed that the acceleration of $\text{He}^{+}$ PUIs in CIRs is substantially more effective than that of $\text{He}^{2+}$ SWIs. The observation that the $\text{He}^{+}/\text{He}^{2+}$ ratio increased linearly with the distance of the CIR source region from the Sun, like the abundance of the PUIs, made this connection abundantly clear (Morris et al. 2001). In a survey of energetic particle events, Kucharek et al. (2003) demonstrated that the high $\text{He}^{+}/\text{He}^{2+}$ ratios observed by Hovestadt et al. (1984) were indeed related to $\text{He}^{+}$ PUIs. Cold solar wind material in magnetic clouds could not be responsible for the high ratios. However, the increased acceleration effectiveness appears to be limited to interstellar PUIs, such as $\text{He}^{+}$ and $\text{Ne}^{+}$. Singly charged $\text{C}^{+}$ and $\text{O}^{+}$, as expected from inner source PUIs, were below the detection threshold of the ACE SEPICA instrument in CIRs (Möbius et al. 2002, 1998a).

#### Distribution Function of PUIs in the Solar Wind Frame

The spectra presented in Figs. 7 and 8 are in the spacecraft reference frame. Thus, the spectra shown in Figs. 7 and 8 can only be considered as a quantity proportional to the particle distribution function integrated over the instrument FOV during the measurement interval. The FOV of the SWICS instrument is large compared to the angular size of PUI distribution in the spacecraft frame. The measurement is sensitively dependent on the direction of the instrument entrance, the solar wind flow direction, and the size of the angular particle distribution, which is particle-speed dependent.

**Fig. 11:**
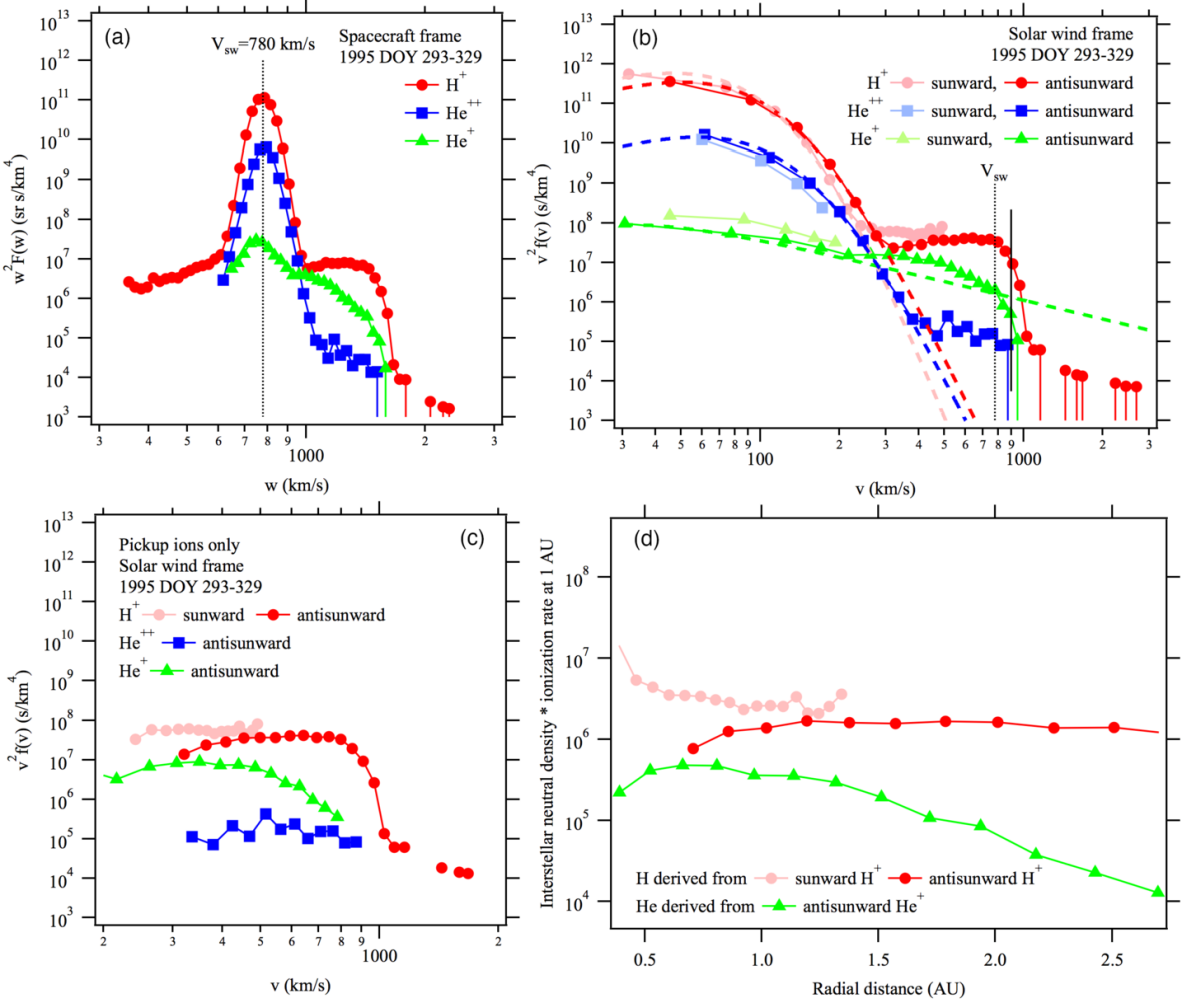
(**a**) Spectra of $\text{H}^{+}$, $\text{He}^{2+}$, and $\text{He}^{+}$ ions measured by Ulysses SWICS as a function of particle speed in the spacecraft reference frame. (**b**) Spectra in the solar wind plasma reference frame assuming that the phase density is only a function of particle speed in the plasma reference frame in each channel of the instrument. In some speed ranges, particles with the same speed are measured by sunward and anti-sunward facing channels separately, which results in two tracks of the distribution function. The low-speed parts of the $\text{H}^{+}$ and $\text{He}^{2+}$ spectra are dominated by the solar wind ions, which can be fit with a kappa distribution as shown by the red and green dashed curves. The low-speed part of the $\text{He}^{+}$ spectrum is fit with a spectrum for inner source PUIs (Schwadron et al. 2000). (**c**) Interstellar PUI spectra after subtraction of fitted spectra of the solar wind $\text{H}^{+}$ and $\text{He}^{2+}$ ions and inner source $\text{He}^{+}$ PUIs. It shows that the interstellar PUIs are not isotropic in the plasma reference frame. (**d**) Radial dependence of interstellar neutral H and He density derived using the PUI distribution formula from Vasyliunas and Siscoe (1976). We note that the radial dependence of He density derived here likely needs a modification because the anisotropy of freshly produced $\text{He}^{+}$ PUIs causes a significant underestimation of the PUI flux

In an attempt to remove as much of the instrumental effects as possible, Zhang et al. (2019) developed a method to transform the SWICS spectra into the solar wind plasma frame of reference. In the plasma frame, it is reasonable to assume that the particle distribution function cannot be very anisotropic (less than an order of magnitude difference in intensity at the same speed, see Fig. 11). The PUI energy density contributes a significant fraction to the total internal energy of the plasma, and thus a highly anisotropic particle distribution would generate a plasma instability. The PUIs can be scattered by turbulence originating in the solar corona or generated by unstable plasma distributions. Encouraged by this realization, Zhang et al. (2019) assumed that the particle distribution within each instrument channel is only a function of particle energy in the solar wind frame, independent of direction, thus allowing them to construct the particle distribution as a function of particle speed in the solar wind frame. This is a reasonable approximation because Ulysses SWICS covers a limited FOV relative to the Sun-spacecraft line for a fixed particle speed in the solar wind frame. Figure 11b shows an example of results obtained from Ulysses SWICS observations in a high-speed solar wind stream at $\sim2.7~\text{au}$ radial distance from the Sun and heliographic latitude $\sim64^{\circ}$ and longitude $\sim240^{\circ}$ from vernal equinox. For comparison, the particle spectra integrated over the instrument FOV in the spacecraft frame are shown in Fig. 11a. The particle speed is shifted through the transformation of the reference frame, but the shape of the distribution function has also changed quite a bit.

The spectra in Fig. 11 are given as speed distributions, $v^{2} f(v)$, a quantity proportional to particle differential flux. The spectra in Fig. 11b contain three distinct populations: SWIs in the range $v<\sim 300~\text{km}\,\text{s}^{-1}$, PUIs between $v=\sim 300$ and $v=\sim 1000~\text{km}\,\text{s}^{-1}$, and suprathermal tail for $v>\sim 1000~\text{km}\,\text{s}^{-1}$. The spectra for $\text{H}^{+}$, $\text{He}^{+}$, and $\text{He}^{2+}$ exhibit a shoulder-like shape until a cutoff speed slightly below $1000~\text{km}\,\text{s}^{-1}$. For $\text{H}^{+}$ and $\text{He}^{2+}$, the lower-speed portions of the spectra are fit with a kappa distribution for SWIs (dotted curves in Fig. 11b). After subtracting the solar wind spectra, we obtain the PUI spectra, as shown in Fig. 11c. However, the low-speed portion of the $\text{He}^{+}$ spectra does not fit well with a kappa distribution function because the function becomes divergent and the derived thermal speed of $\text{He}^{+}$ is much larger than for solar wind $\text{H}^{+}$ and $\text{He}^{2+}$. This result suggests that the low-speed $\text{He}^{+}$ flux is not of solar wind origin. Rather, they could be $\text{He}^{+}$ PUIs from an inner source. This part of the spectrum is fit with a distribution derived from Schwadron et al. (2000), and the result is presented as the dashed green curve in Fig. 11b. After the inner PUI source contribution is subtracted from the $\text{He}^{+}$ spectrum, we obtain the interstellar $\text{He}^{+}$ PUI spectrum, shown by the green points in Fig. 11c.

The spectral shapes of the three ion species look different, indicative of the radial density variation of particle sources that produce these PUIs. Using Eq. (1), we can calculate the density of interstellar neutral H and He as a function of radial distance along the Ulysses-Sun line at the time. Since we do not know the ionization rate at that time, the results shown in Fig. 11d are given as a product of interstellar neutral density times their ionization rate at 1 au.

In the solar wind frame, the PUI cutoff speed is approximately the solar wind speed, i.e., $V_{\mathrm{inj}} = \vert \boldsymbol{V}_{\mathrm{SW}} - \boldsymbol{V}_{\text{H/He}} \vert \cong V_{\mathrm{SW}}$. Careful examination of the rapid decreases of PUI intensity around $\sim900~\text{km}\,\text{s}^{-1}$ suggests that the PUI cutoff speed is slightly higher than the solar wind speed of $780~\text{km}\,\text{s}^{-1}$. However, the difference of $120~\text{km}\,\text{s}^{-1}$ in the cutoff speed cannot be explained by the fluctuation of solar wind bulk speed, the spacecraft motion, or interstellar neutral atom velocity along the Ulysses orbit. It means that some mechanism has accelerated the PUIs. Since there is no shock found during the time period, the likely mechanism is through stochastic acceleration either by Alfvenic or compressible turbulence in the solar wind (Chalov et al. 2003; Le Roux and Ptuskin 1998; Zhang and Lee 2013).

There is a slight anisotropy of the $\text{H}^{+}$ PUI distribution in the solar wind frame. We use different shades of colors to indicate the intensity of particles moving in directions along or against the solar wind flow. The lighter shaded points are from those channels that see particles moving towards the Sun but convect with the solar wind flow to bring them into the instrument as low-energy particles. Apparently, $\text{H}^{+}$ PUIs moving toward the Sun have higher intensity levels than those moving away from the Sun by a factor of 2 to 3. This phenomenon is consistent with the anisotropy of $\text{H}^{+}$ PUI reported by Gloeckler et al. (1995). The anisotropy is only seen in these PUIs up to half of the solar wind speed, simply because the instrument cannot measure those particles with speeds below a certain energy threshold in the spacecraft frame. The low-speed particles in the solar wind frame are cooled PUIs that originated closer to the Sun and subsequently convected outward to the spacecraft. It appears that those with lower speed or more cooled PUIs have a larger anisotropy than those with $\sim1/2$ the solar wind speed. This behavior is consistent with what was predicted by Schwadron et al. (2000) using a transport model of a bi-hemispherical PUI distribution. The model predicts that the anisotropy would increase again for freshly born PUIs with speed equal to the solar wind speed in the plasma frame.

## Observations of Interstellar PUIs in the Outer Heliosphere

From the earliest studies of the origin and production of interstellar PUIs in the solar wind, it was predicted that they would constitute a significant fraction of the solar wind plasma pressure at large distances from the Sun and approaching the HTS. However, it becomes significantly difficult to detect interstellar PUIs at increasingly larger distances from the Sun due to the radial expansion of the solar wind plasma and decrease in plasma density with distance proportional to $r^{-2}$. An instrument with a sufficiently large geometric factor is required to measure interstellar PUIs in the outer heliosphere. While evidence of interstellar $\text{H}^{+}$ PUIs was seen in Pioneer 10 and 11 observations out to $\sim15~\text{au}$ from the Sun with simultaneous measurements of waves attributed to the PUIs (Intriligator et al. 1996; Mihalov and Gazis 1998), the signatures were relatively weak and the coverage in energy was limited. To date, the only direct measurement of the interstellar PUI distribution beyond $\sim10~\text{au}$ was achieved with the Solar Wind Around Pluto (SWAP) (McComas et al. 2008) instrument aboard the New Horizons mission (Stern 2008; Young et al. 2008).

The SWAP instrument is specifically designed to measure interstellar $\text{H}^{+}$ PUIs in the solar wind with a high signal-to-noise ratio due to its wide aperture design, rotation about a Sun-pointed axis, and coincidence detection method. Launched in 2006, the primary goal of SWAP was to observe the plasma environment in the solar wind around Pluto and Kuiper Belt Objects. The SWAP instrument took measurements of the plasma environment during the New Horizons’ flyby of Pluto on 2015 July 14 (Bagenal et al. 2016; McComas et al. 2016) and continues taking measurements as New Horizons travels farther away from the Sun on its extended mission to observe the outer heliosphere. During the course of its operation, SWAP has provided the only in situ observations of interstellar PUI distributions beyond $\sim10~\text{au}$ from the Sun (McComas et al. 2010; Randol et al. 2013, 2012), quantifying the nonthermal distribution of $\text{H}^{+}$ PUIs in the solar wind plasma and their radial trend from $\sim20~\text{au}$ where the instrument was permitted to stay on nearly continuously, out to roughly halfway to the HTS (McComas et al. 2021). This section reviews the history of SWAP observations of interstellar PUIs, their distribution function, radial trends, interactions with interplanetary shocks, and their properties extrapolated to the HTS.

### SWAP Instrumentation for PUI Measurements in the Outer Heliosphere

SWAP uses a top-hat shaped ESA to maximize angular coverage of the sky when measuring the highly nonthermal PUI population. Ions are detected in the energy/charge range $\sim0.021$ and 7.8 keV/q with FWHM resolution $\Delta E/E = 0.085$ (McComas et al. 2008). PUIs can enter SWAP’s aperture from a large region of the sky (instantaneously $10^{\circ}\times276^{\circ}$), and as the New Horizons spacecraft spins about a Sun-pointed axis, nearly the entire sky is viewed. SWAP uses two channel electron multipliers (CEMs) to make coincidence measurements for improved signal-to-noise determinations of the interstellar PUI signal. SWAP takes measurements across its energy/charge range every 64 sec, where PUI measurements are collected at a 24 hr cadence to fit within the very limited telemetry allowed by the mission. SWAP is also equipped with a retarding potential analyzer (RPA) used to deflect particles below a certain energy/charge threshold in the inner heliosphere, where the fluxes were dangerously high; however, use of the RPA was discontinued after the Jupiter flyby.

**Fig. 12:**
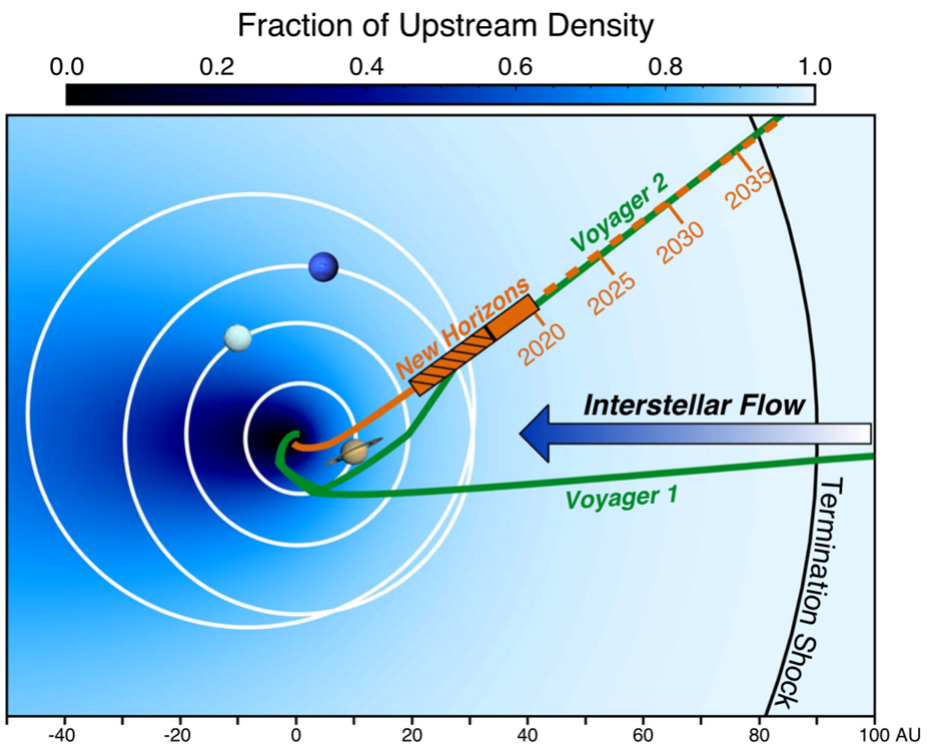
Illustration of New Horizons’ trajectory through the heliosphere (orange). The latest SWAP data release includes measurements taken over the time covered by the orange boxes. Trajectories of Voyagers 1 and 2 are also shown. From McComas et al. (2021)

Figure 12 shows the trajectory of New Horizons projected onto the ecliptic plane. After its encounter with Jupiter in 2007, New Horizons is traveling at approximately the same ecliptic longitude as Voyager 2, but close to the ecliptic plane. Currently $\sim50~\text{au}$ from the Sun, it is projected that New Horizons will reach the HTS (nominally expected to be $\sim90~\text{au}$ from the Sun) in approximately 15 yr. Whether all instruments will remain operational until then is unknown and depends on the power production of the radioisotope thermoelectric generator. It is expected that SWAP will be operational for most of its trip to the HTS, which may for the first time provide direct measurements of PUI mediation and acceleration at the HTS, as has been predicted by numerous theoretical, modeling, and data analysis studies (Ariad and Gedalin 2013; Decker et al. 2008; Giacalone et al. 2021; Giacalone and Decker 2010; Kumar et al. 2018; Lee et al. 1996; Lembège and Yang 2016; Matsukiyo and Scholer 2014; Richardson et al. 2008a; Yang et al. 2015; Zank et al. 2010, 1996; Zilbersher and Gedalin 1997; Zirnstein et al. 2021b).

### Interstellar $\text{H}^{+}$ PUI Distribution Function

**Fig. 13:**
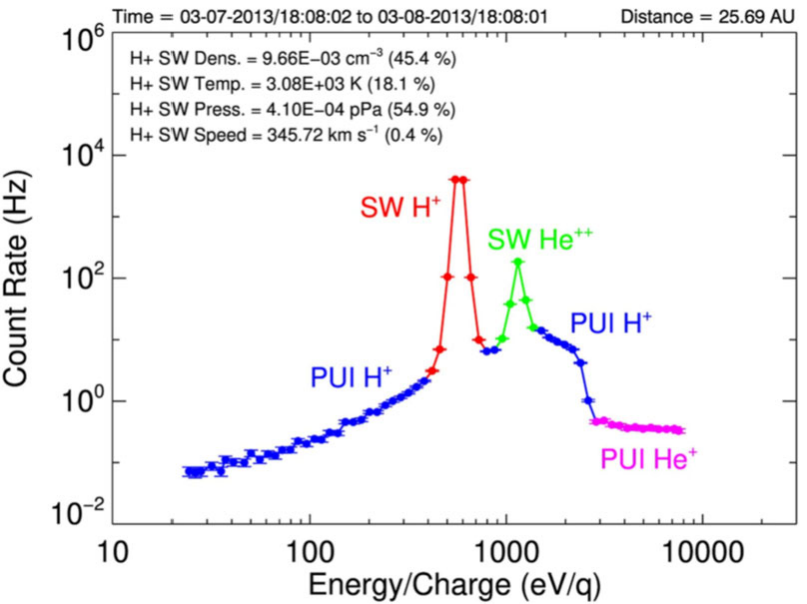
SWAP observations at 25.7 au from the Sun. The spectrum is color-coded to show the primary source of the counts. The blue spectra identify the $\text{H}^{+}$ PUI observations that are fit with a generalized filled-shell distribution. From McComas et al. (2017b). © AAS. Reproduced with permission

Several studies examined the interstellar $\text{H}^{+}$ PUI distribution in the solar wind with a sparse set of observations from SWAP between $\sim10$ and 22 au (McComas et al. 2010; Randol et al. 2013, 2012), first quantifying the plasma pressure contributed by PUIs to the solar wind far beyond the ionization cavity. Beyond 22 au, SWAP began operating nearly continuously, providing long range detail of the radial trends of interstellar PUIs (McComas et al. 2017b). Figure 13 shows an example of a count rate sweep accumulated over 24 hrs at 25.7 au from the Sun, where $\text{H}^{+}$ SWIs, $\text{H}^{2+}$ alpha particles, $\text{H}^{+}$ PUIs, and $\text{He}^{+}$ PUIs are shown in different colors. Using Eq. (1) and the ‘cold’ model for interstellar neutral H density, given as (Thomas 1978)

4 $$ n_{\mathrm{H}} ( r,w, \theta ) = n_{\mathrm{H},\mathrm{TS}} \exp \biggl( - \frac{\lambda}{r} \frac{\theta}{\sin \theta} w^{-3/2} \biggr), $$

where $n_{\mathrm{H},\mathrm{TS}}$ is the interstellar neutral H density at the HTS, McComas et al. (2017b) analyzed the density and thermal properties over $\sim22\text{--}38~\text{au}$ from the Sun. They found that PUIs are not consistent with the physical interpretation of the Vasyliunas and Siscoe (1976) distribution. In particular, the values for the local H ionization rate, $\beta _{0}$, and ionization cavity, $\lambda $, derived from fitting Eq. (1) using a forward model of SWAP count rates yielded unrealistically large values. The authors concluded that although Eq. (1) can be used to fit to SWAP PUI observations, the interstellar PUI distribution is physically different from the representation of Eq. (1). Following this analysis, Swaczyna et al. (2020) applied a generalized formulation of Eqs. (1) and (4) to SWAP observations and found better agreement with the ionization rate and cavity parameters that yielded physically meaningful values. This formula is given by (Chen et al. 2014; McComas et al. 2021)

5 $$ \begin{aligned} f_{\mathrm{PUI}} ( \boldsymbol{r}, w,\theta ) &= \frac{\alpha S( \boldsymbol{r},w)}{4\pi} \frac{\beta _{0} r_{0}^{2}}{r V_{\mathrm{sw}} V_{\mathrm{inj}}^{3}} w^{\alpha -3} n_{\mathrm{H}} ( r,w, \theta ,\alpha ) \Theta ( 1-w ), \\ n_{\mathrm{H}} ( r,w, \theta ,\alpha ) &= n_{\mathrm{H},\mathrm{TS}} \exp \biggl( - \frac{\lambda}{r} \frac{\theta}{ \sin \theta} w^{-\alpha} \biggr), \end{aligned} $$

where $\alpha $ is the cooling index, $n_{\mathrm{H},\mathrm{TS}}$ is the interstellar neutral H density at the HTS (Swaczyna et al. 2020), and $S(\boldsymbol{r},w)$ is the survival probability of PUIs from their point of creation to the point of observation. In the inner heliosphere, $S(\boldsymbol{r},w)$ is close to 1 since re-neutralization of PUIs is negligible, but decreases farther from the Sun. Equation (5) is a more generalized form of Eq. (1) by allowing a representation for the heating or nonadiabatic cooling of PUIs.

**Fig. 14:**
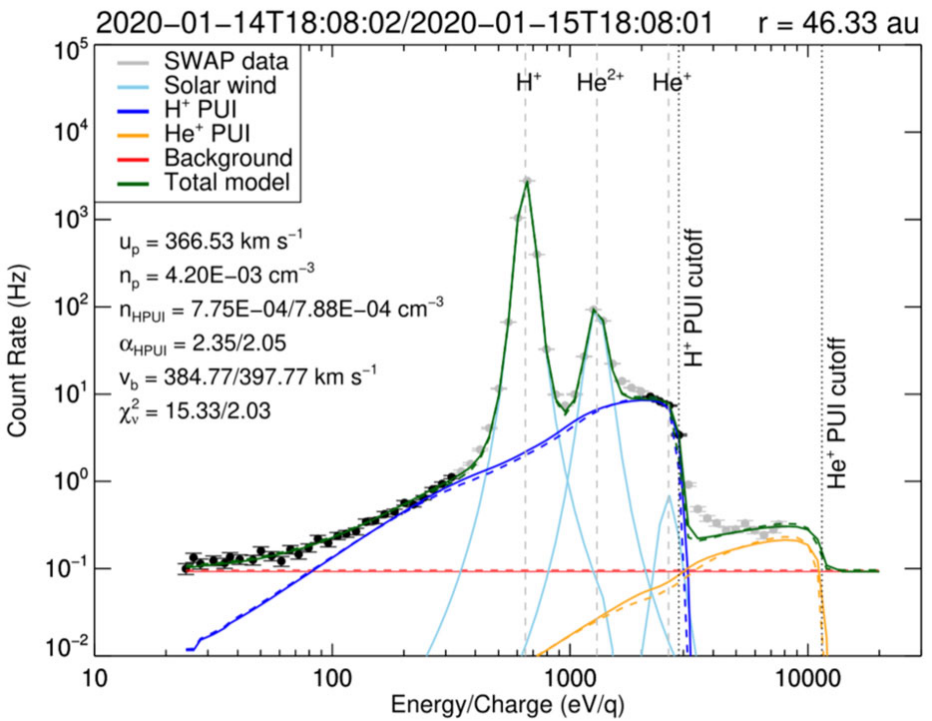
SWAP observations at 46.33 au from the Sun. Solar wind $\text{H}^{+}$ and $\text{He}^{2+}$ are fit with kappa functions, $\text{H}^{+}$ PUIs are fit with a generalized filled shell (Eq. (5)). From McComas et al. (2021)

McComas et al. (2021) reanalyzed the SWAP PUI data set and extended them to farther distances from the Sun between $\sim22$ and 47 au using Eq. (5). They found a significant improvement in the statistical fitting of the fit parameters in Eq. (5) to the PUI observations. An example of the SWAP PUI observations fit by Eq. (5) is shown in Fig. 14, at 46 au from the Sun. The $\text{H}^{+}$ PUI shell cutoff is visible at $\sim 2 V_{\mathrm{SW}}$, and the $\text{He}^{+}$ PUI shelf at higher energies is also measured though measurement energy range generally does not extend up to the $\text{He}^{+}$ PUI shell cutoff. A detailed description of the fitting to the other ion components within the solar wind is given by McComas et al. (2021). These authors also improved fitting to the $\text{H}^{+}$ PUI cutoff by allowing the cutoff speed to be a free fit parameter. SWAP observations reveal that the PUI cutoff speed can slightly vary from the relative speed of the solar wind and interstellar neutrals due to, e.g., particle interactions with shocks/compressions or turbulence.

As discussed in Sects. 2.4.1 and 2.4.2, there is considerable evidence for the presence of PUI-generated magnetic fluctuations in the outer heliosphere. It is reasonable to expect that, far beyond the interstellar H ionization cavity ($>20~\text{au}$), $\text{H}^{+}$ PUIs not only hold a significant amount of the internal plasma pressure but they also are responsible for generating magnetic fluctuations that heat the thermal SW ions (Isenberg 2005; Matthaeus et al. 1999; Pine et al. 2020; Smith et al. 2001; Zank et al. 2018). It is not completely clear, however, how much intrinsic vs. PUI-generated magnetic fluctuations influence the scattering and potential heating of the PUI distribution. As we have described thus far, SWAP observations currently suggest that PUIs measured beyond 20 au are well-represented by a generalized, isotropic filled-shell distribution. The generalization represents a deviation from adiabatic cooling, which is summarized in Sect. 3.3.

### Nonadiabatic Heating in the Distant Solar Wind

**Fig. 15:**
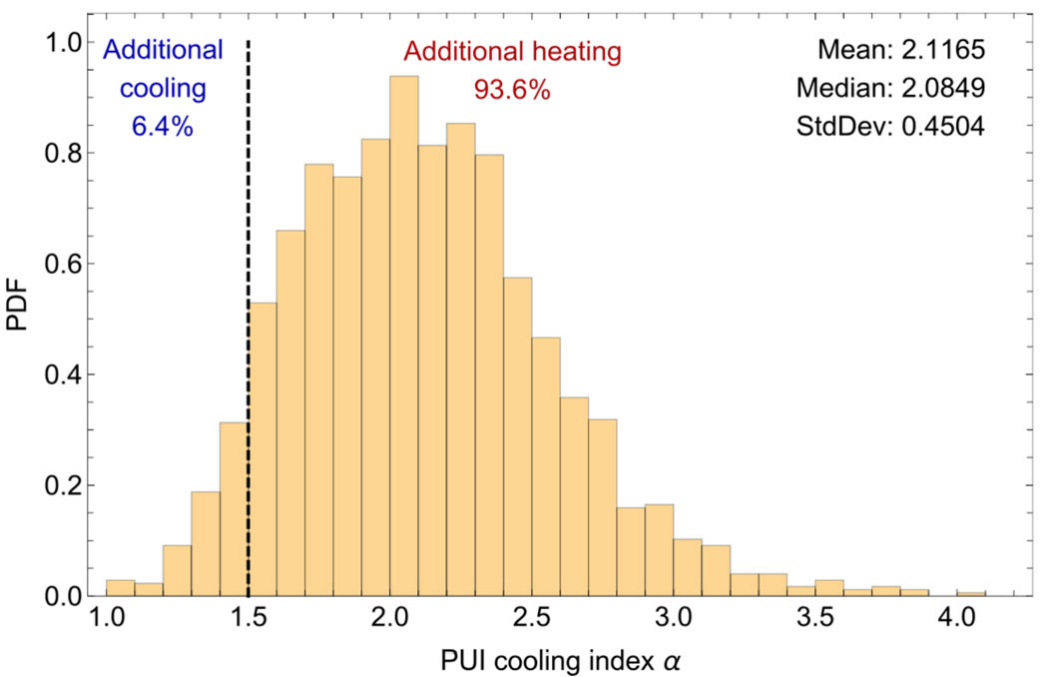
Histogram of PUI distribution cooling index from SWAP observations between $\sim22$ and 47 au. From McComas et al. (2021)

The new methods of fitting to SWAP $\text{H}^{+}$ PUI measurements revealed that interstellar PUIs are nonadiabatically heated in the solar wind for the majority of the time (McComas et al. 2021). Figure 15 shows a histogram of the PUI cooling index over the entire SWAP dataset. The PUI cooling index $\alpha$, whose value is 1.5 for the case of adiabatic cooling in the expanding solar wind, is observed to be greater than 1.5 for 93.6% of the measurements. The deviation from 1.5 is quite large, where the mean of $\alpha $ is 2.1 and the standard deviation 0.45. These measurements strongly suggest that PUIs are experiencing additional heating in the solar wind as they propagate to the outer heliosphere. McComas et al. (2021) looked into whether compressions and shocks may be responsible for the nonadiabatic heating of PUIs by performing a superposed epoch analysis of a few dozen shocks observed by SWAP. They found that while the PUI density and temperature experienced jumps coinciding with the shock jumps in solar wind speed, the cooling index did increase near shocks but delayed by a few days. The PUI cooling index gradually rises for $\sim1$ week after the passage of the shock and eventually decreases over about a week after that. It is not clear why the PUI cooling index behaves this way, but McComas et al. (2021) posited that since the cooling index is coupled to the PUI cutoff speed, the delayed reaction of the rise and fall of the cooling index may be due to an enhancement of freshly-injected PUIs in the higher density solar wind downstream of the shocks.

We note that the nonadiabatic nature of interstellar PUI heating and cooling was also observed in ACE SWICS observations near 1 au. Chen et al. (2013) found that while the cooling index of $\text{He}^{+}$ PUIs between 1999 and 2010 was on average close to 1.5, there were significant deviations to lower and higher values that appeared to be correlated with the solar cycle and solar activity such as in solar wind compression regions. Chen et al. (2014) refined the possible influences on $\text{He}^{+}$ PUI cooling indices by determining that the effects of electron impact ionization, which become significant close to the Sun, are negligible on the cooling index. Thus, ACE SWICS observations of the $\text{He}^{+}$ PUIs also support the idea that the nonadiabatic cooling of PUIs in the solar wind are likely driven by particle interactions with shocks or compressions.

### Preferential Heating at Interplanetary Shocks

**Fig. 16:**
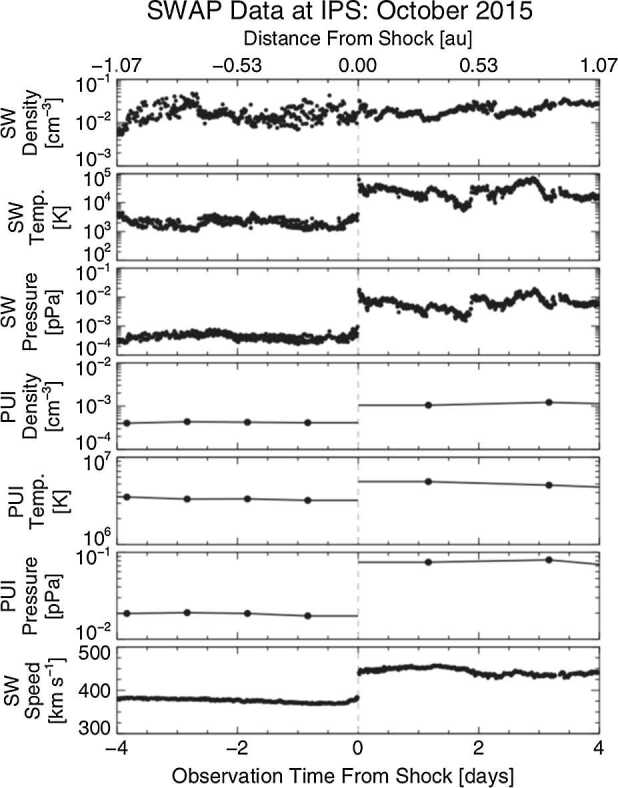
SWAP observations at an interplanetary shock $\sim34~\text{au}$ from the Sun. SWI measurements are made at a higher cadence (10 min) than PUI measurements (1 day). From Zirnstein et al. (2018). © APS. Reproduced with permission

**Fig. 17:**
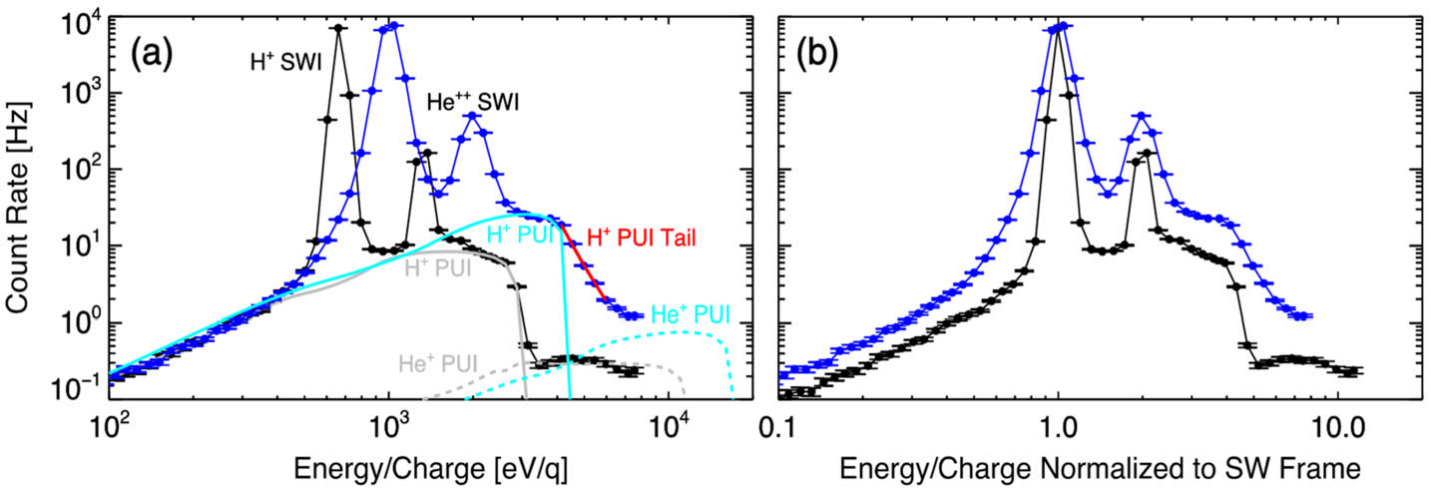
SWAP count rates before (black) and after (blue) the shock binned over 24 hrs. Fits to the PUI filled shell upstream and downstream are shown as gray and cyan, and a fit to the downstream PUI tail is shown in red. From Zirnstein et al. (2018). © APS. Reproduced with permission

The behavior of interstellar $\text{H}^{+}$ PUIs at an interplanetary shock was studied in detail by Zirnstein et al. (2018) for a relatively strong shock at $\sim34~\text{au}$ from the Sun (Fig. 16). SWAP observed an abrupt increase in SW speed, density, and temperature on 2015 October 5 which, without the measurements of magnetic field, is the indicator used to identify the presence of shocks or compressions at New Horizons. Using SWAP measurements of the PUI density compression across the shock, Zirnstein et al. (2018) estimated that the shock compression ratio is $\sim2.5$ when including only the filled shell of PUIs. The PUI filled shell increased in temperature by $\sim65\%$ across the shock. Interestingly, SWAP observed a suprathermal PUI tail downstream of the shock that lasted for a few days before disappearing (red curve in Fig. 17). A forward model fit of a power-law to the suprathermal tail revealed a slope of $v^{-9.7}$. The tail represents $\sim15\%$ of the total downstream PUI density, and its temperature is $\sim1.1\times10^{7}~\text{K}$. The relatively steep PUI tail is likely a result of preferential acceleration at the shock, consistent with PUI reflection at the cross-shock potential (Zank et al. 2010, 1996).

The shock epoch analysis from McComas et al. (2021) found that $\text{H}^{+}$ PUIs contain the majority of the energy flux of the plasma downstream of interplanetary shocks ($\sim70\%$ of SWIs + PUIs + magnetic energy), similar to the findings of Zirnstein et al. (2018). These observations are a direct indication of the preferential heating of PUIs at interplanetary shocks in the outer heliosphere, and likely play a more significant role at mediating the HTS.

### Radial Trends and Extrapolation to the Termination Shock

**Fig. 18:**
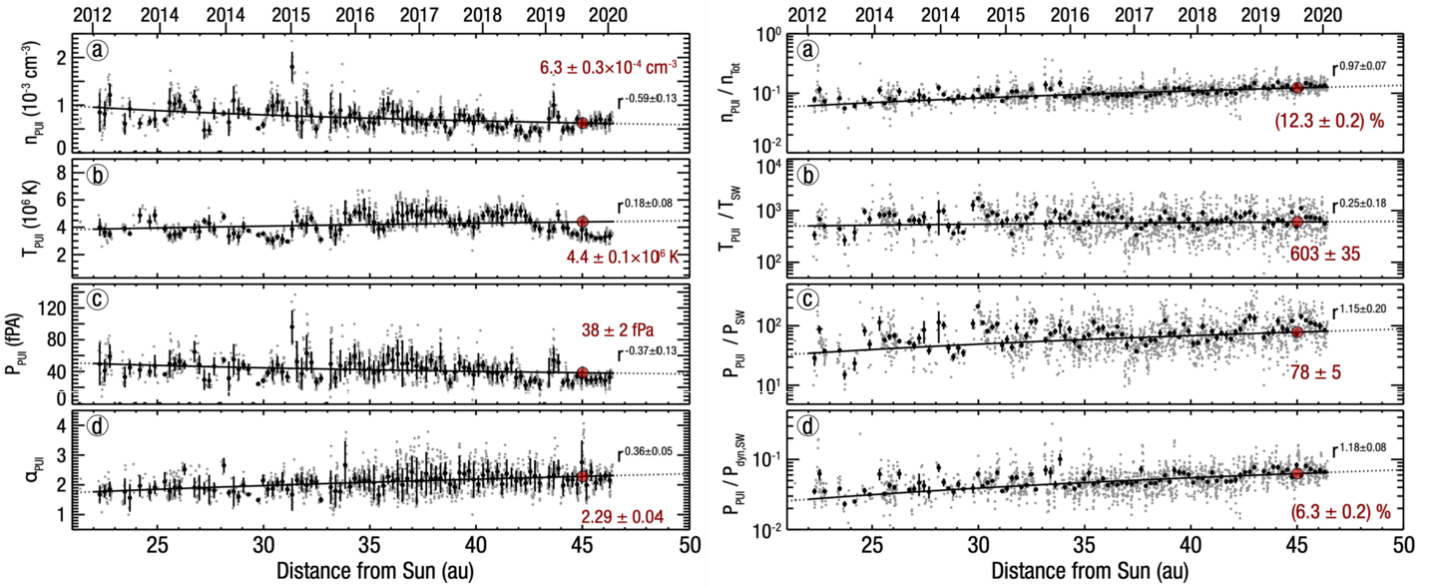
(left) SWAP PUI measurements between 22 and 47 au (gray data points). Power-law functions are fit to solar rotation-averaged values (black data with uncertainties), whose uncertainties represent the time variability within each time-averaged sample. The power law fits are shown in black and nominal values of PUI parameters at 45 au in red. (right) Ratios of daily-averaged parameters (gray) and ratios binned over solar rotation (black). Power-law fits to the ratios are shown in black. From McComas et al. (2021)

With New Horizons having traveled almost 50 au from the Sun, we are able to quantify radial trends of the PUI distribution with greater accuracy than ever. Analyses of PUI radial trends were updated by McComas et al. (2021) and then extrapolated to 90 au to provide predictions for the PUI distribution function at the HTS. Figure 18 shows radial trends fit to SWAP PUI measurements between 22 and 47 au. The left panels show fits to the PUI density, temperature, pressure, and cooling index and their “fiducial” values at 45 au for use by the heliospheric modeling and data analysis community. At 45 au from the Sun, PUIs hold most of the internal plasma pressure and constitute $\sim12\%$ of the proton density. As the solar wind continues to propagate to the outer heliosphere, more PUIs will be picked up and their density relative to the core SWIs will grow.

**Fig. 19:**
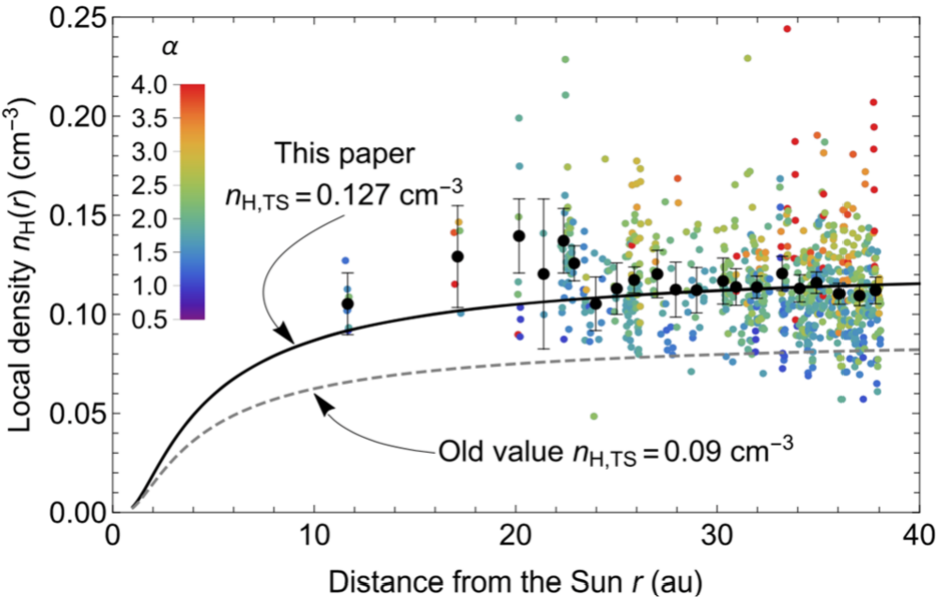
Density of interstellar neutral H as a function of distance from the Sun derived from SWAP observations. Extrapolation of H density to the upwind HTS reveals a density value that is $\sim40\%$ larger than previous estimates. From Swaczyna et al. (2020). © AAS. Reproduced with permission

Since New Horizons is far beyond the ionization cavity of interstellar neutral H, SWAP measures a larger amount of interstellar PUIs compared to measurements closer to Earth. This enabled Swaczyna et al. (2020) to use SWAP measurements of interstellar PUIs to accurately derive the density of interstellar H atoms local to SWAP and extrapolate the density to the HTS. They derived a density of $0.127\pm0.015~\text{cm}^{-3}$ at the HTS, which is $\sim40\%$ larger than previous consensus values (Fig. 19). Swaczyna et al. (2020) determined that previous values of neutral H density using spacecraft measurements closer to Earth (Bzowski et al. 2009) may be inaccurate due to the low $\text{H}^{+}$ PUI density inside the ionization cavity. Swaczyna et al. (2020) also found that the neutral H density derived from Voyager measurements (Richardson et al. 2008b) can be in agreement with New Horizons SWAP when using a more accurate charge exchange cross section. A higher interstellar neutral H density has significant implications on the outer heliosphere, because it changes the rate of charge exchange and the number of ENAs created in the heliosheath. This may partially explain a long-standing issue with models of heliosheath ENA flux, which appear to underestimate IBEX observations of ENAs by a factor of $\sim2$ (Baliukin et al. 2020; Kornbleuth et al. 2020; Zirnstein et al. 2017).

The accumulation and advection of interstellar PUIs with the solar wind is also indirectly observed in the slowing of the solar wind plasma (Richardson et al. 2008b), which is a sign of mass-loading of the solar wind by PUIs (Szegö et al. 2000). Elliott et al. (2019) analyzed SWAP measurements of solar wind speed at New Horizons and found that the solar wind slowed down by $\sim5\text{--}7\%$ between 1 au and the solar wind plasma observed by SWAP at 30–43 au, in agreement with the expected rate of mass loading by ionized interstellar neutral H atoms. This finding was achievable by comparing SWAP observations at 30–43 au with ACE and STEREO observations at 1 au at times when they were aligned, expanding on the direct observational evidence for the dynamical effect of the interstellar neutral pickup process on the solar wind plasma.

SWAP observations can be used to predict realistic values for PUI density and temperature at the HTS. This is particularly useful for studies of particle acceleration at the HTS, and to better understand Voyager observations near the HTS without direct observations of PUIs. However, there is considerable variability in the SWAP measurements due to solar wind shocks, compressions, corotating interactions regions, etc., that negatively influence radial trend fitting. To minimize these effects, McComas et al. (2021) fit radial trends to the ratios of density, temperature, and pressure, shown in the rights panels of Fig. 18, which show less variability over distance and time. Extrapolating the ratio fits to 90 au, the PUI density ratio ${n_{\mathrm{H},\mathrm{PUI}}} / { ( n_{\mathrm{H},\mathrm{PUI}} + n_{\mathrm{H},\mathrm{SWI}} )} =24\%$ and the PUI-to-SW dynamic pressure ratio ${P_{\mathrm{H},\mathrm{PUI}}} / {P_{\mathrm{SW},\mathrm{Dyn}}} =14\%$. Thus, at the HTS, PUIs hold a significant amount of the plasma energy and will strongly mediate the shock interaction. Preferential $\text{H}^{+}$ PUI heating at interplanetary shocks has been observed by Ulysses SWICS (Gloeckler et al. 1994a) and New Horizons SWAP (McComas et al. 2021; Zirnstein et al. 2018) and the effects of shock mediation at the HTS has been seen indirectly by Voyager 2 (Richardson et al. 2008a).

## Conclusions and Future Perspectives

The ubiquitous presence of interstellar PUIs in the solar wind has been apparent since the earliest studies of solar wind transport through the solar system and outer heliosphere and its interaction with the partially ionized interstellar medium. The role of PUIs in mediating heliospheric shocks, their contribution to the solar wind plasma pressure, and their function as the primary source for heliospheric ENA fluxes have been confirmed by a wealth of spacecraft observations.

Theoretical predictions for the distribution of interstellar PUIs in the solar wind have existed for decades, and an array of spacecraft near Earth have made observations of PUIs ever since (Sects. 2–3). The most notable discoveries have been the nonthermal nature of the PUI distributions, the capability of using interstellar PUI measurements as a diagnostic of the interstellar medium properties, and their preferential heating at interplanetary shocks. One of the most defining predictions of the PUI distribution incorporated in the solar wind is their dominating contribution to the internal plasma pressure that increases with distance from the Sun, implying their growing contribution to the plasma pressure and mediation of shocks in the outer heliosphere.

**Fig. 20:**
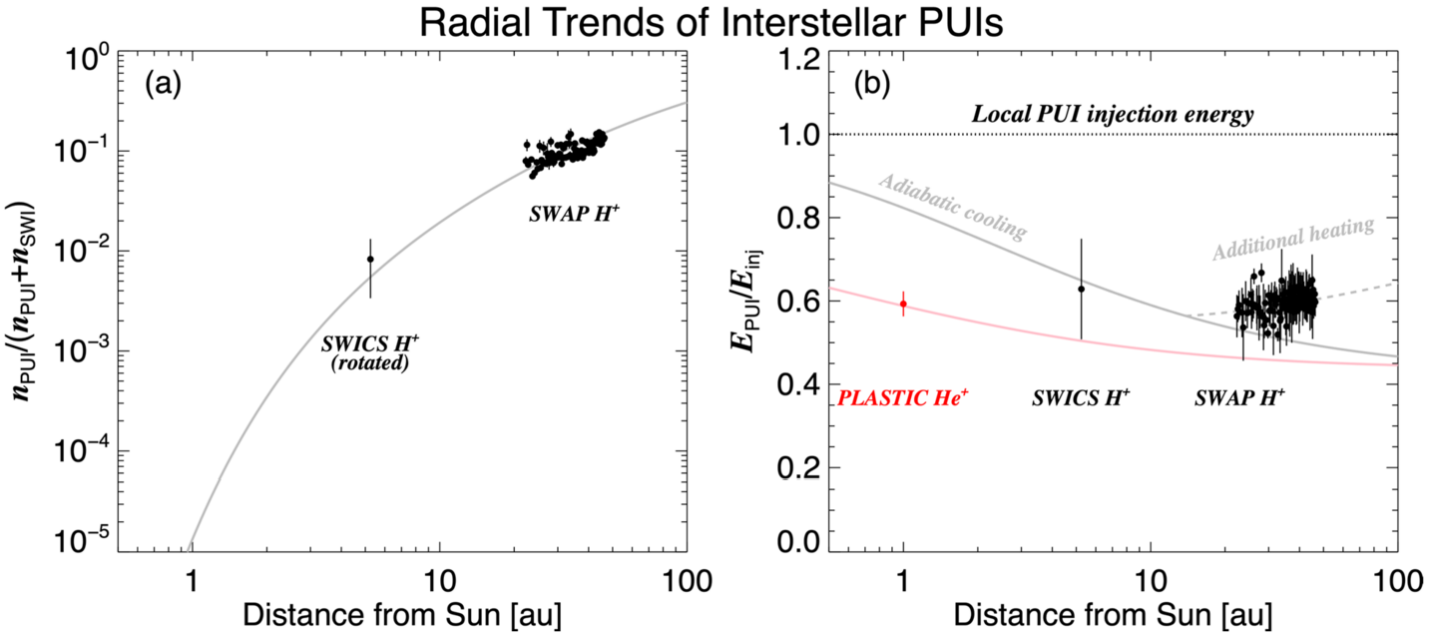
Interstellar $\text{H}^{+}$ (black) and $\text{He}^{+}$ (red) PUI observations made by STEREO PLASTIC at 1 au, Ulysses SWICS at 5 au, and New Horizons’ SWAP at 22–47 au. (**a**) Ratio of interstellar $\text{H}^{+}$ PUI density to total proton density (SWI + PUI). (**b**) Ratio of interstellar PUI thermal energy to local injection energy. Models of the density and energy ratios are also shown. New Horizons and Ulysses observations are made at different ecliptic longitudes and reflect different ionization cavity sizes; therefore, we rotate SWICS $\text{H}^{+}$ PUI densities towards the longitude of New Horizons’ trajectory

To demonstrate the radial trends of interstellar PUI density and temperature, we present a small collection of multi-spacecraft measurements of interstellar PUIs in Fig. 20. We show the ratio of $\text{H}^{+}$ PUI density to total proton density, ${n_{\mathrm{H},\mathrm{PUI}}} / { ( n_{\mathrm{H},\mathrm{PUI}} + n_{\mathrm{H},\mathrm{SWI}} )}$, in Fig. 20a and the ratio of $\text{H}^{+}$ or $\text{He}^{+}$ PUI internal energy to the local PUI injection energy, ${E_{\mathrm{PUI}}} / {E_{\mathrm{inj}}}$, in Fig. 20b. We also show model expectations of the density and energy ratios, which are calculated from the zeroth and second order moment integrations of Eq. (5) (by ignoring the PUI survival probability term $S( \boldsymbol{r},w)$, which is typically close to 1). The $\text{H}^{+}$ PUI density and temperature can be derived analytically for a cold interstellar neutral H gas, under the assumption that the forces of gravity and radiation pressure balance, as

6 $$ \begin{aligned} n_{\mathrm{H},\mathrm{PUI}} ( \boldsymbol{r}, \theta ) &= \frac{\beta _{0} r_{0}^{2}}{r V_{\mathrm{SW}}} n_{\mathrm{H},\mathrm{TS}} \bigl[ \exp ( - \Lambda ) - \Lambda E_{1} ( \Lambda ) \bigr], \\ T_{\mathrm{H},\mathrm{PUI}} ( \boldsymbol{r}, \theta ) &= \frac{m_{\mathrm{H}}}{3 n_{\mathrm{H},\mathrm{PUI}} k_{\mathrm{B}}} \frac{\beta _{0} r_{0}^{2} V_{\mathrm{inj}}^{2}}{r V_{\mathrm{SW}}} n_{\mathrm{H},\mathrm{TS}} E_{2+ {2} / {\alpha}} ( \Lambda ), \\ \Lambda &= \frac{\lambda _{\mathrm{H}}}{r} \frac{\theta}{\sin \theta}, \end{aligned} $$

where $E_{y} ( x )$ is the exponential integral. For $\text{He}^{+}$ PUIs, the gravitational force on interstellar He atoms cannot be neglected. Constraining our analysis to observations near the LISM upwind direction ($\theta \cong 0$), the density and temperature of $\text{He}^{+}$ PUIs is calculated numerically as

7 $$ \begin{aligned} n_{\mathrm{He},\mathrm{PUI}} ( r ) &= \frac{\alpha \beta _{0} r_{0}^{2}}{r V_{\mathrm{sw}}} \int _{0}^{1} w^{\alpha -1} n_{\mathrm{He}} ( r,w,\alpha ) dw, \\ T_{\mathrm{He},\mathrm{PUI}} ( r ) &= \frac{m_{\mathrm{He}}}{3 n_{\mathrm{He},\mathrm{PUI}} k_{\mathrm{B}}} \frac{\alpha \beta _{0} r_{0}^{2} V_{\mathrm{inj}}^{2}}{r V_{\mathrm{sw}}} \int _{0}^{1} w^{\alpha +1} n_{\mathrm{He}} ( r,w,\alpha ) dw, \\ n_{\mathrm{He}} ( r,w,\alpha ) &= n_{\mathrm{He},\mathrm{TS}} \exp \biggl[ - \lambda _{\mathrm{He}} \biggl( \frac{V_{\mathrm{He},\mathrm{ISN}}^{2}}{GM} \biggr) \biggl( \sqrt{1+ {2} / { \biggl( \frac{V_{\mathrm{He},\mathrm{ISN}}^{2}}{GM} r \biggr)}} -1 \biggr) w^{-\alpha} \biggr]. \end{aligned} $$

Here we again assume a cold neutral He interstellar gas (Chen et al. 2013), which is a valid approximation near the LISM upwind direction (Fahr 1971; Thomas 1978). The density of interstellar neutral He far from the Sun $n_{\mathrm{He},\mathrm{TS}} =0.015~\text{cm}^{-3}$ and the He ionization cavity size $\lambda _{\mathrm{He}} =0.5~\text{au}$ (Gloeckler et al. 2004).

As shown in Fig. 20a, the relative density of $\text{H}^{+}$ PUIs grows exponentially with distance, starting below 1% within 10 au of the Sun and reaching $>10\%$ at distances covered by New Horizons SWAP observations halfway to the HTS. This trend is primarily driven by the decline in interstellar neutral H density close to the Sun as it becomes ionized by charge exchange in the solar wind. The uncertainty of the $\text{H}^{+}$ PUI density observed by Ulysses SWICS at 5 au, representing the standard deviation of measurements over a 2-month period in 2003 when Ulysses was near the ecliptic plane, is partially due to variability in the ionization rate of interstellar neutral H in slow vs. fast solar wind streams over short time scales. Farther from the Sun, however, the variability in the solar wind speed is reduced due to interactions between fast and slow solar wind streams, leading to a less variable PUI density ratio (Elliott et al. 2019, 2016). The model curve presented in Fig. 20a assumes an average ionization rate consistent with New Horizons SWAP measurements, and an ionization cavity size of 4 au (Swaczyna et al. 2020).

The interstellar PUI energy or temperature in the solar wind plasma frame as a function of distance from the Sun is shown in Fig. 20b for a selection of measurements from Ulysses SWICS, New Horizons SWAP, and STEREO PLASTIC. The PUI measurements are presented as the ratio of PUI energy to local injection energy, i.e., ${E_{\mathrm{PUI}}} / {E_{\mathrm{inj}}} = { \langle v_{\mathrm{PUI}}^{2} \rangle} / {V_{\mathrm{inj}}^{2}}$, where $\langle v_{\mathrm{PUI}}^{2} \rangle $ is the mean squared PUI speed in the plasma frame, and $V_{\mathrm{inj}} = \vert \boldsymbol{V}_{\mathrm{SW}} - \boldsymbol{V}_{\mathrm{H}/\mathrm{He},\mathrm{ISN}} \vert $ is the local PUI injection speed for $\text{H}^{+}$ or $\text{He}^{+}$ PUIs, where we assume $V_{\mathrm{H},\mathrm{ISN}} =22~\text{km}\,\text{s}^{-1}$ (Lallement et al. 2005) and $V_{\mathrm{He},\mathrm{ISN}} =25.4~\text{km}\,\text{s}^{-1}$ (McComas et al. 2015). It is expected that ${E_{\mathrm{PUI}}} / {E_{\mathrm{inj}}}$ should always be less than 1, since at distance $r$ from the Sun the PUI distribution is comprised of PUIs injected at different points in the history of the plasma parcel (see, e.g., Fig. 4 in Swaczyna et al. 2020), with older PUIs having experienced more cooling in the radially expanding solar wind. Therefore, in the absence of suprathermal PUI tails and significant slowing of the solar wind (estimated to be $<10\%$ halfway to the HTS; Elliott et al. 2019), the energy of the PUI filled shell distribution will be lower than the local solar wind speed, with increasingly smaller energy ratio farther from the Sun. Ulysses SWICS observations of $\text{H}^{+}$ PUIs are consistent with adiabatic cooling (see model curve derived from Eq. (6) for $\alpha =1.5$), though the standard deviation is significant. Farther from the Sun, SWAP observations reveal a PUI cooling index larger than 1.5, suggesting an additional source of heating. The dashed curve in Fig. 20b is derived from a power law fit to the PUI cooling index from McComas et al. (2021). It is not clear from SWICS observations if $\text{H}^{+}$ PUIs exhibit nonadiabatic cooling within 5 au of the Sun, but the dominance of the PUI thermal pressure beyond $\sim20~\text{au}$ likely effects how PUIs interact with shocks and compressions at New Horizons.

While it is difficult to derive absolute $\text{H}^{+}$ and $\text{He}^{+}$ PUI densities near 1 au due to their scarcity, it is possible to determine the energy ratio of $\text{He}^{+}$ PUIs in the solar wind frame. Figure 20b shows the energy ratio of $\text{He}^{+}$ PUIs derived from STEREO PLASTIC at 1 au. The measurement is a time average of data collected within $\pm5^{\circ}$ longitude of the LISM upwind direction, and uncertainty representing the 1-$\sigma $ standard deviation. As one can see, the energy ratio of $\text{He}^{+}$ PUIs is lower on average to that of $\text{H}^{+}$ PUIs. This is primarily due to the lower rate of ionization of interstellar neutral He, where more neutral He atoms can travel closer to the Sun before becoming ionized. We show a model of the $\text{He}^{+}$ PUI energy ratio assuming adiabatic cooling as the red curve, based on Eq. (7) assuming a He ionization cavity size of 0.5 au. The smaller ionization cavity implies that more $\text{He}^{+}$ PUIs at distance $r$ had originated from locations closer to the Sun, had experienced more cooling in the expanding solar wind, and thus yield a colder PUI distribution. Far from the ionization cavities of H and He, it is expected that any adiabatically cooled $\text{H}^{+}$ or $\text{He}^{+}$ PUI distributions will approach similar energy ratios in the solar wind ($\sim0.45$ at $r > 50~\text{au}$), but this likely is not true if $\text{H}^{+}$ and $\text{He}^{+}$ are heated differently at shocks and compressions in the outer heliosphere. We note that a study of $\text{He}^{+}$ PUI measurements made by Cassini CHEMS between 1 and 9 au from the Sun revealed an unusual increase in suprathermal $\text{He}^{+}$ PUI intensity with distance from the Sun (Hill et al. 2009) during quiet times in the solar wind. Hill et al. (2009) suggested that stochastic acceleration and an unknown velocity-dependent acceleration mechanism may be responsible for heating of $\text{He}^{+}$ PUIs in the solar wind.

We note that the PLASTIC and SWICS measurements presented in Fig. 20 are culled for times when the angle between the IMF vector and solar wind velocity is close to $90^{\circ}$. Although New Horizons is not equipped with a magnetometer, it is expected that at such large distances from the Sun the mean magnetic field is nearly perpendicular to the solar wind velocity (Bagenal et al. 2015; Burlaga and Ness 1996).

Extrapolating the SWAP measurements of PUI properties to the HTS provides values of PUI density, temperature, and cooling index that can be used by modelers of particle acceleration at the HTS. The nonadiabatic cooling index of the PUI distribution extrapolated to the HTS ($\sim2.9$) (McComas et al. 2021) is important to consider in these studies, because PUIs at low speeds in the shock frame, or those close to the PUI shell cutoff moving towards the Sun in the plasma frame, will most likely experience preferential reflection and energization at the shock. This has direct implications for heliospheric ENA measurements by IBEX and the upcoming Interstellar Mapping and Acceleration Probe (IMAP) mission (McComas et al. 2018). PUIs accelerated at the HTS are the source of ENAs produced in the inner heliosheath of the heliosphere observed, i.e., the GDF (Schwadron et al. 2014; Zirnstein et al. 2017) as well as some of the secondary ENAs from outside the heliopause that form the IBEX Ribbon (Heerikhuisen et al. 2010; McComas et al. 2009a; Zirnstein et al. 2015).

The New Horizons spacecraft is projected to reach 90 au in the mid-2030s. If SWAP is still operating by the time New Horizons reaches the HTS, it will provide the first in situ observation of interstellar PUI acceleration at the HTS, which is important for understanding global ENA observations, the seed population to diffusive shock acceleration, and pressure balance between the solar wind and interstellar medium plasmas.

Looking into the future of near-Earth observations, PUI and suprathermal tail studies in the inner heliosphere will receive a boost with the launch of IMAP in 2025 (McComas et al. 2018). The spacecraft will carry two instruments that measure the PUI distributions over the entire hemisphere in the solar direction, the Solar Wind And Pickup Ion (SWAPI) instrument for $\text{He}^{+}$ PUIs and the Compact Dual Ion Composition Experiment (CoDICE) for $\text{He}^{+}$ through $\text{Ne}^{+}$ PUIs, thus enabling an in-depth analysis of their behavior in the solar wind. SWAPI will observe the $\text{He}^{+}$ PUI cutoff speed and the start of any rollover or tail of the $\text{He}^{+}$ PUIs, while CoDICE covers the suprathermal tails at higher energies. The collective measurements by SWAPI and CoDICE will reveal in greater detail the distribution of PUIs in the quiet solar wind and upstream and downstream of interplanetary shocks at high temporal and energy resolution, allowing us to better understand the role of preferential PUI heating and energization at shocks. Measurements of ENA emissions from the outer heliosphere will also be enhanced by the IMAP-Lo, IMAP-Hi, and IMAP-Ultra imagers, which will improve our understanding of PUI distributions in the heliosheath.
